# A randomized-controlled neurofeedback trial in adult attention-deficit/hyperactivity disorder

**DOI:** 10.1038/s41598-021-95928-1

**Published:** 2021-08-19

**Authors:** Beatrix Barth, Kerstin Mayer-Carius, Ute Strehl, Sarah N. Wyckoff, Florian B. Haeussinger, Andreas J. Fallgatter, Ann-Christine Ehlis

**Affiliations:** 1 Psychophysiology and Optical Imaging, Department of Psychiatry and Psychotherapy, University of Tübingen, Calwerstr. 14, 72076 Tübingen, Germany; 2 LEAD Graduate School and Research Network, University of Tübingen, Gartenstr. 29, 72074 Tübingen, Germany; 3 Department of Psychiatry and Psychotherapy, Tübingen Center for Mental Health (TüCMH), University of Tübingen, Calwerstr. 14, 72076 Tübingen, Germany; 4 Institute for Medical Psychology and Behavioural Neurobiology, University of Tübingen, Silcherstr. 5, 72076 Tübingen, Germany; 5 Werner Reichardt Centre for Integrative Neuroscience (CIN), University of Tübingen, Otfried-Müller-Str. 25, 72076 Tübingen, Germany; 6 Present Address: Faculty of Sport Science, Institute of Sport Psychology and Physical Education, Leipzig University, Jahnallee 59, 04109 Leipzig, Germany; 7 Present Address: Division of Pediatric Neurology, Barrow Neurological Institute at Phoenix Children’s Hospital, Phoenix, AZ USA

**Keywords:** Cognitive neuroscience, ADHD, Psychology

## Abstract

Attention-deficit/hyperactivity disorder (ADHD) is a childhood onset disorder persisting into adulthood for a large proportion of cases. Neurofeedback (NF) has shown promising results in children with ADHD, but randomized controlled trials in adults with ADHD are scarce. We aimed to compare slow cortical potential (SCP)- and functional near-infrared spectroscopy (fNIRS) NF to a semi-active electromyography biofeedback (EMG-BF) control condition regarding changes in symptoms and the impact of learning success, as well as changes in neurophysiological parameters in an adult ADHD population. Patients were randomly assigned to SCP-NF (*n* = 26), fNIRS-NF (*n* = 21) or EMG-BF (*n* = 20). Outcome parameters were assessed over 30 training sessions (pre, intermediate, post) and at 6-months follow-up (FU) including 3 booster sessions. EEG was recorded during two auditory Go/NoGo paradigms assessing the P300 and contingent negative variation (CNV). fNIRS measurements were conducted during an n-back- as well as a Go/NoGo task. All three groups showed equally significant symptom improvements suggesting placebo- or non-specific effects on the primary outcome measure. Only when differentiating between learners and non-learners, fNIRS learners displayed stronger reduction of ADHD global scores compared to SCP non-learners at FU, and fNIRS learners showed specifically low impulsivity ratings. 30.8% in the SCP-NF and 61.9% of participants in the fNIRS-NF learned to regulate the respective NF target parameter. We conclude that some adults with ADHD learn to regulate SCP amplitudes and especially prefrontal hemodynamic activity during NF. We did not find any significant differences in outcome between groups when looking at the whole sample. When evaluating learners only, they demonstrate superior effects as compared to non-learners, which suggests specific effects in addition to non-specific effects of NF when learning occurs.

## Introduction

Attention-deficit/hyperactivity disorder (ADHD) is a childhood onset disorder which is characterized by persistent, age-inappropriate levels of inattention and/or hyperactive-impulsive behaviors in multiple settings^1^. Depending on individual symptom patterns, we can classify three presentations of ADHD, namely a combined presentation, a predominantly inattentive presentation and a predominantly hyperactive-impulsive presentation (DSM-5^1^). In about 65% of diagnosed cases, ADHD symptoms persist into adulthood^2,3^ with estimated prevalence rates of 1.4% to 3.6%^4^. In adults, ADHD is accompanied by disorganization, impairments in occupational life and/or difficulties on a social level^5^ as well as psychiatric disorders such as affective disorders or substance use disorders^6^. Treatment options for ADHD commonly involve pharmacological treatment usually accompanied by psychological, educational and social interventions^7^. Pharmacotherapy entails various advantages, e.g., effects eventuate more or less immediately. However, uncertainty about long-term effects^8^, side effects^9^, as well as a non-negligible proportion of non-responders^7^ substantiate the need for alternative treatments. A plethora of studies revealed that a hypoactivation of the prefrontal cortex (PFC) underlies many of the above-described deficits^10^. The right inferior frontal cortex has repeatedly been reported to exhibit hypoactivation in ADHD wherein the right inferior frontal gyrus has been specifically associated with motor response inhibition^11,12^. The PFC plays an essential role in the regulation of executive functioning such as attentional control, impulse control, planning or working memory^13–15^. On an electrophysiological level, impaired regulation of slow cortical potentials (SCP) in children^16^ as well as adults with ADHD has been shown^17^. SCPs are a specific type of event-related potentials (ERP) characterized by slow electrical positive or negative shifts that reflect an increase or decrease in cortical excitation thresholds^16^. The contingent negative variation (CNV) is a slow negative potential developing between conditional and imperative stimuli reflecting anticipatory and preparatory processes^18^. A reduced CNV amplitude has been found in adults with ADHD compared to healthy controls^19^. The P300 component is elicited roughly 300 ms after a behaviorally relevant stimulus and reflects attentional resource allocation due to updating of target representations^20^. Reduced P300 amplitudes have been repeatedly reported in adults with ADHD during target detection^19,21^ which is hypothesized to reflect impaired attentional resource allocation.

Thus, a treatment that directly addresses these neurocognitive deficits might constitute a way to effectively manage ADHD symptoms. In neurofeedback (NF), participants can learn to voluntarily regulate a certain aspect of their brain physiology through repeated practice and continuous feedback. One of the potential advantages of NF over pharmacological treatment might be the stability of improvements beyond the intervention period^22^. Various studies conducted SCP-NF in children and adolescents with ADHD (cf.,^23^) and found relatively large effect sizes regarding the general improvement of clinical symptoms; however, with blinded assessments, these effect sizes considerably diminished raising some doubts concerning the specificity of the effects^24,25^. In contrast, there are few NF studies using neuroimaging technologies measuring hemodynamic brain activity, namely functional magnetic resonance imaging (fMRI) in adolescents^26,27^ and adults with ADHD^28^, as well as functional near-infrared spectroscopy (fNIRS) in children with ADHD^29^. Previously, we implemented fNIRS-NF in healthy subjects (proof-of-concept) as well as different groups of patients with psychiatric conditions (for review, see^30^). Compared to fMRI, fNIRS is relatively insensitive to motion artifacts and measurements can be performed in rather natural settings. However, as compared to the temporal resolution attainable in EEG, the speed of operation with fNIRS is limited due to the nature of the metabolic response underlying the signal.

As studies on NF in adults with ADHD are scarce, we tried to fill the gap and investigated the efficacy, specificity, learning and long-term stability of SCP- and fNIRS-NF in comparison to a semi-active EMG biofeedback (BF) control. We analyzed changes in amplitude, and differentiation of task-specific activation over the training course in feedback and transfer conditions. Based on the differentiation of task-specific activation by the end of the training in transfer trials, we categorized participants into learners and non-learners to scrutinize differences concerning training outcome on ADHD symptoms. We aimed to investigate changes in ADHD symptoms as well as comorbid symptoms. Furthermore, we sought to investigate changes in P300 and CNV amplitudes as well as prefrontal hemodynamic responses during a working memory and Go/NoGo task. To assess the magnitude of non-specific effects, we used a semi-active control BF training (EMG-BF). Regarding hypotheses, we expected (1) both active neurofeedback trainings (SCP and fNIRS) to be superior in terms of symptom reductions compared to the semi-active control condition (EMG-BF); (2) comparable changes in symptomatology in fNIRS- compared to SCP neurofeedback and greater changes in comparison to EMG feedback after 30 sessions of training; (3) outcomes to be stable over 6 months follow-up period; (4) adults with ADHD to be able to learn to control the respective target parameter during neurofeedback; (5) primary outcomes to be more pronounced in learners compared to non-learners; (6) changes in specific ERP- and fNIRS parameters related to improved cognitive preparation, attention, response inhibition and working memory.

## Methods

This study was conducted at the Institute of Medical Psychology and Behavioral Neurobiology (SCP-NF, EEG assessments) and the Department of Psychiatry and Psychotherapy (fNIRS-NF, EMG-BF, fNIRS assessments) at the University of Tübingen. The project planning started in June 2010 and the last data was assessed in December 2015. The trial (DRKS00006767) was approved by the local Ethics Committee for the Medical Department, University of Tübingen, Germany, Ethics vote number: 434/2010B01. Written informed consent was given by all participants. This study is registered with the German Registry of Clinical Trials: DRKS00006767, date of registration: October 8th, 2014.

### Participants

Participants were recruited from the University of Tübingen student population as well as non-student adults through university mailing lists, flyers, newspaper advertisement, registered local doctors, and support groups. We conducted randomization of a total of 84 participants in two steps comprising a blockwise randomization, and a pairwise randomization regarding age and IQ (assessed by Culture Fair Intelligence Test Scale 2-revised, CFT-20-R^31^). We specified a grouping order (SCP = 1; fNIRS = 2; EMG = 3). A participant that didn’t match group 1 was allocated to group 2, and if not group 2, then group 3 by the assessors. Starting point for the next participant was then group 2 etcetera. Neither blinding of participants nor blinding of assessors to the training condition was possible due to the different setups (SCP, EMG and fNIRS) inherent to the different feedback methods that made it obvious which was the target parameter^32^.

**Figure 1:**
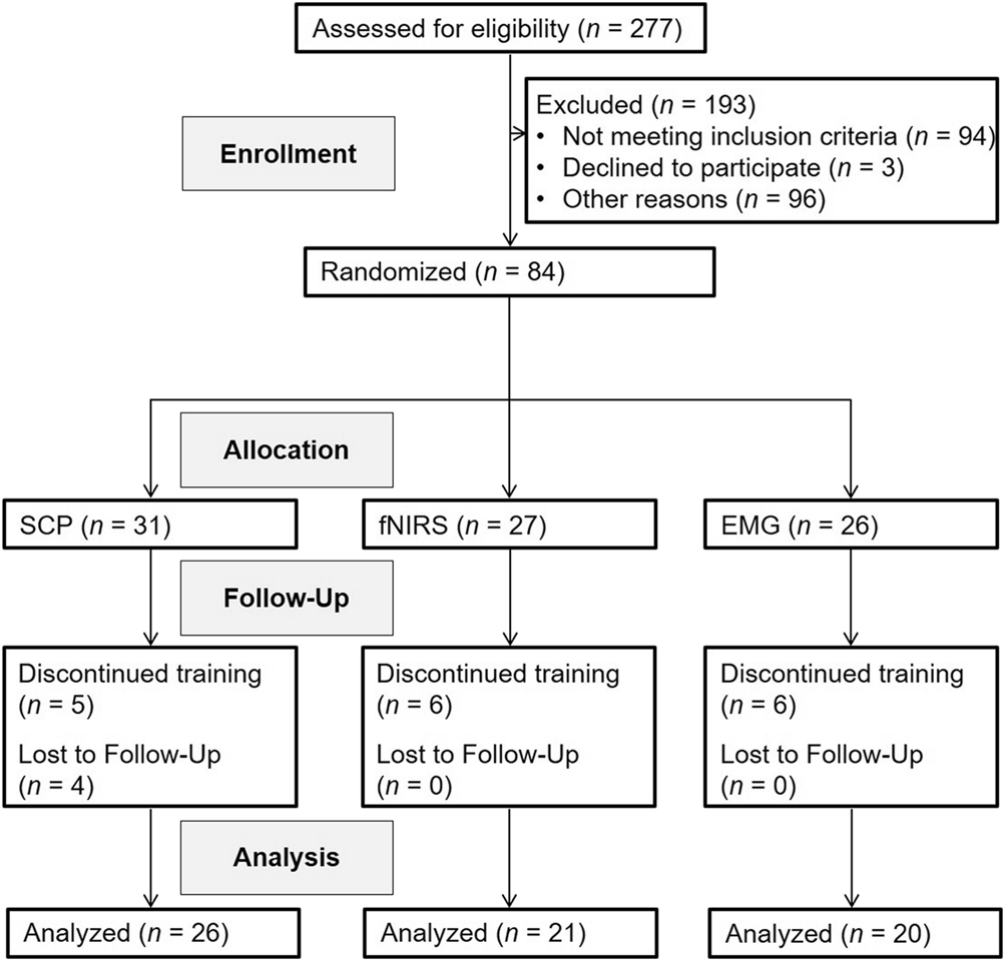
Diagram of participant flow.

The subjects' inclusion and exclusion in this analysis are shown in Fig. 1. Five subjects in the SCP-, six in the fNIRS-NF and six in the EMG-BF dropped out but were replaced to ensure sufficient test power. Besides fulfilling diagnostic criteria for ADHD (without differentiation of presentations, i.e., diagnostic subtypes), further inclusion criteria comprised age of at least 18 years and intelligence quotient over 80 (CFT-20-R). We used self-report scales for retrospective evaluation of childhood ADHD symptoms (short version of the German Wender Utah Rating Scale, WURS-K cutoff ≥ 30^33,34^) and symptoms in adulthood (German version of the ADHD self-rating scale, ADHS-SB cutoff ≥ 18^35,36^), third-party questionnaires for childhood and adulthood symptoms (“Fragebogen zur Erfassung von ADHS im Erwachsenenalter, frühere/aktuelle Probleme - Fremdbeurteilung”, FEA-FFB/ FEA-AFB^37^) as well as a diagnostic interview (Wender Reimherr Interview, WRI^36^) conducted by a psychologist^32^. To exclude comorbid psychiatric disorders (except for moderate depression), the German version of the Structured Clinical Interview for DSM-IV axis I and II disorders (SCID I and II^38^) was conducted by a psychologist as well^32^. A neurological disease or ongoing psychotherapy were exclusion criteria while pharmacotherapy was permitted with constant dosage^32^. Six patients in the SCP-NF, seven in the fNIRS-NF and five in the EMG-BF received pharmacological treatment (methylphenidate) at a constant dosage which was assessed by questionnaire every fifth session. Group characteristics are listed in Table 1. To maintain training motivation, participants paid a deposit between 50 and 120 Euro depending on their financial situation, which was refunded after study completion.

**Table 1:** Sample characteristics.

Characteristic	SCP (*n* = 26)	fNIRS (*n* = 21)	EMG (*n* = 20)	Test statistic; effect size (group comparison)
Age, years, mean ± SD	33.62 ± 10.24 [range 22–53]	31.24 ± 9.97 [range 18–50]	33.65 ± 12.64 [range 18–56]	*Kruskal–Wallis* *H* = 0.93; *n.s*.; *d*_Cohen_ = 0.261
Sex, male/female, no	14/12	14/7	14/6	*Kruskal–Wallis* *H* = 1.45; *n.s*.; *d*_Cohen_ = 0.187
Handedness, right/left, no	22/3^#^	17/3^#^	16/4	*Kruskal–Wallis* *H* = 0.54; *p* = .763; *d*_Cohen_ = 0.306
IQ, mean ± SD*	106.46 ± 19.82	112.62 ± 17.68	110.50 ± 13.72	*F*(2,64) = 0.76; *n.s*; *η*_*p*_^2^ = 0.023
WURS-K, mean ± SD	36.46 ± 10.60	44.81 ± 11.08	42.10 ± 8.58	*F*(2,64) = 4.13; ***p* = .021**; *η*_*p*_^2^ = 0.114 Post-hoc Analysis: SCP versus fNIRS: *t*(45) = − 2.63; ***p* = .012**
ADHS-SB Global, mean ± SD	30.31 ± 6.70	34.83 ± 7.40	32.20 ± 6.19	*F*(2,64) = 2.59; *n.s*; *η*_*p*_^2^ = 0.075
ADHS-SB Inattention, mean ± SD	15.46 ± 4.61	17.71 ± 5.30	16.80 ± 3.65	*F*(2,64) = 1.44; *n.s*; *η*_*p*_^2^ = 0.043
ADHS-SB Hyperactivity, mean ± SD	7.88 ± 2.18	9.00 ± 3.52	8.25 ± 2.95	*F*(2,64) = 0.88; *n.s*; *η*_*p*_^2^ = 0.027
ADHS-SB Impulsivity, mean ± SD	6.96 ± 2.63	8.12 ± 2.39	7.15 ± 2.46	*F*(2,64) = 1.36; *n.s*; *η*_*p*_^2^ = 0.041
WRI Global, mean ± SD	38.50 ± 7.05	41.76 ± 7.34	40.15 ± 6.52	*F*(2,64) = 1.27; *n.s*; *η*_*p*_^2^ = 0.038
WRI Inattention, mean ± SD	8.58 ± 1.42	8.71 ± 1.49	8.70 ± 1.45	*F*(2,64) = 0.07; *n.s*; *η*_*p*_^2^ = 0.002
WRI Hyperactivity, mean ± SD	4.69 ± 1.16	5.38 ± 1.28	4.90 ± 1.02	*F*(2,64) = 2.10; *n.s*; *η*_*p*_^2^ = 0.061
WRI Impulsivity, mean ± SD	6.85 ± 2.28	6.95 ± 2.25	6.70 ± 1.49	*F*(2,64) = 0.08; *n.s*; *η*_*p*_^2^ = 0.002
FEA current, mean ± SD^§^	23.78 ± 10.76	24.37 ± 11.46	24.00 ± 10.95	*F*(2,64) = 0.02; *n.s*; *η*_*p*_^2^ = 0.001
FEA past, mean ± SD^+^	28.34 ± 5.24	30.96 ± 7.69	30.44 ± 9.51	*F*(2,64) = 0.82; *n.s*; *η*_*p*_^2^ = 0.025
BDI, mean ± SD	11.31 ± 7.31	11.71 ± 8.88	12.75 ± 7.45	*F*(2,64) = 0.21; *n.s*; *η*_*p*_^2^ = 0.007
Medication (MPH/ATX/DEX)	6 (6/0/0)	7 (5/1/1)	5 (5/0/0)	*Kruskal–Wallis* *H* = 0.66; *n.s*; *d*_Cohen_ = 0.292

*Note.* *ADHS-SB* = German ADHD self-rating scale for symptoms in adulthood^35,36^; *ATX* = Atomoxetine; *BDI* = Beck Depression Inventory^47^; *d*_Cohen_ = , effect size Cohen’s d; *DEX* = Dexamphetamine; *MPH* = Methylphenidate; *n.s*. = not significant; *WRI* = Wender-Reimherr-Interview^35,36^; *WURS-K* = short version of the German Wender Utah Rating Scale for ADHD childhood symptoms^33,34^; *SD* = standard deviation; *η*_*p*_^2^ = partial Eta squared.

*IQ was assessed based on the Culture Fair Intelligence Test, Scale 2-revised (CFT-20-R)^31^.

^#^Information missing for 1 participant.

^+^Information missing for 32 participants.

^§^Information missing for 25 participants.

### Study procedure

Study design, methods and data analysis plan are described in detail in the trial protocol^32^ and are based on the protocol by Holtmann and colleagues^39^. The initial screening was conducted via phone, and questionnaires were mailed to check for the inclusion criteria. Along with the mailed questionnaires, detailed information material and the informed consent form was sent. The final extensive ADHD diagnostic assessment was scheduled if inclusion criteria were met in the questionnaires^32^.

Clinical, EEG-(quantitative EEG, ERPs in cognitive tasks), and fNIRS assessments (changes in oxygenated hemoglobin concentration elicited by executive functioning tasks) were carried out at pre-intervention, after half of the sessions (i.e., 15 sessions), post-training (i.e., 30 sessions), and 6 months after the end of the training (follow-up, FU). Pre-, intermediate-, post- and FU assessments were conducted without medication (for a period of 24 h)^32^.

Participants were trained one to maximum five times per week for a total of 30 sessions. After 15 sessions there was a three-week break with instructions to further practice the so far acquired self-regulation skills in everyday life. To support this “home-work”, participants received a small card with their chosen feedback object imprinted as a reminder as well as a CD playing a video of the transfer trials^32^.

In total, mean training duration of the 30 sessions was 26.64 weeks (*SD* = 11.07; Min/Max = 14.57–55.86) for SCP-NF, 28.32 weeks (*SD* = 8.87; Min/Max = 12.29–49.14) for fNIRS-NF and 27.81 weeks (*SD* = 9.00; Min/Max = 9.00–51.00) for EMG-BF with no significant differences between groups (*F*(2,64) = 0.18; *p* = 0.834). Six months after training, a FU assessment and three booster sessions evaluated the stability of acquired regulation ability as well as neurophysiological and clinical outcome variables. In SCP and EMG, each session lasted about 1 h, including preparation time. In fNIRS, each session lasted about 40 min. To generalize the newly acquired self-regulation skills into daily routines, 25% of trials were implemented as a “transfer block” in which no visual feedback was presented but participants received reinforcement following the trial in case they had regulated in the desired direction^32^. For the rest of the trials, visual feedback of brain/muscle activity was provided by means of a moving object on the screen which participants could select beforehand^32^. In the reward phase, participants were visually reinforced by the symbol of a sun presented on the screen immediately following successful trials (SCP and EMG: at least 2 s of the second half of the trial; fNIRS: at least 7 s of the last 15 s regulation in the desired direction)^32^. Additionally, the therapist gave positive verbal feedback. Participants were not given explicit strategies, but were given broad suggestions about how regulation might work.

#### Slow cortical potential neurofeedback

SCP feedback was conducted with the DC-EEG-neurofeedback and biofeedback system THERA PRAX utilizing a DC-EEG- and bio signal amplifier (neuroConn GmbH, Ilmenau, Germany) with a monopolar setting (Cz referenced against mastoid A2 with a ground electrode on mastoid A1)^32^. Ag/AgCl ring electrodes were used on all sites. Four electrodes recorded vertical and horizontal eye movements. The device conducted an online artifact correction for eye movements using a calibration file that was generated at the beginning of each session. Likewise, the system identified signal changes above 200 μV, e.g., caused by movements. In case of an artifact, the trial was aborted and repeated^32^.

**Figure 2:**
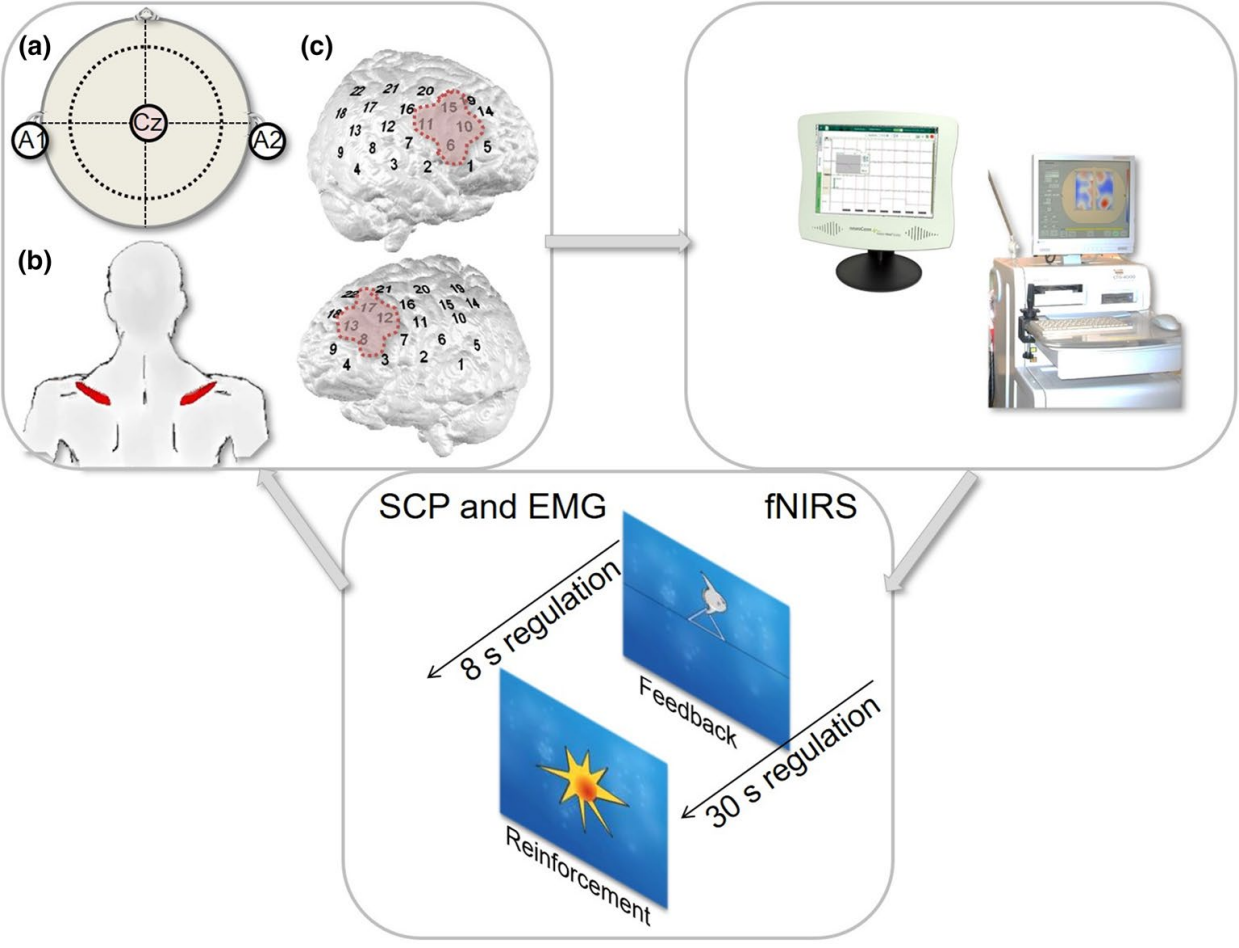
Experimental setup for (**a**) EEG-, (**b**) EMG- and (**c**) fNIRS feedback.

Each SCP session comprised four runs (8 min each) of 40 trials with each trial lasting 12 s and consisting of three phases: baseline (seconds 0 to 2), regulation with visual feedback (e.g., a moon, seconds 2 to 10), and reinforcement in case of successful regulation (seconds 10 to 12)^32^. Following the baseline, subjects were presented with a triangle pointing to the top of the screen requiring brain “activation” (i.e., electrically negative shifts) or a triangle pointing to the bottom prompting participants to “deactivate” their brain (i.e., electrically positive shifts)^32^. In all sessions, 50% activation and 50% deactivation trials were randomly presented (Fig. 2)**.**

#### Functional near-infrared spectroscopy neurofeedback

fNIRS signals were recorded by means of the ETG-4000 continuous wave system (Hitachi Medical Co., Japan) which was linked to the THERA PRAX and a personal computer. fNIRS data were provided from the ETG-4000 to the personal computer via TCP/IP protocol for further online processing using MATLAB R2011 to calculate the input signal for the THERA PRAX^29^. Participants sat in front of a monitor in a dark and sound-attenuated room and received visual feedback about changes in oxygenated hemoglobin (O_2_Hb) over left and right prefrontal areas^32^. To cover frontal sites on both hemispheres, we used two 3 × 5 optode probe sets (consisting of seven photodetectors and eight light emitters, respectively; i.e., 22 channels) resulting in a total amount of 44 channels (Fig. 2). The inter optode distance was 3 cm. Probe sets were oriented based on the international 10–20 system for electrode placement^40^. Fpz was marked as mid-point and additionally T3 and T4, respectively, as positions to place the rearmost channel in the lowest line of the respective probe set^29^. Sampling rate was 10 Hz. The fNIRS feedback signal was computed online using a common average reference to deal with global artifacts. For each data point during the regulation, mean O_2_Hb changes of four frontal channels per probe set were calculated^29^. In a next step, the average activity of all channels on the respective probe set was subtracted. Finally, the resulting O_2_Hb amplitudes for each probe set were averaged^29^.

Every session consisted of three blocks and 32 min NF in total. fNIRS-NF included two feedback blocks of 12 trials, each block lasting 12 min, separated by an 8 min transfer block of 8 regulation trials. At the beginning of each session, a 10-s baseline was recorded. A regulation trial lasted 30 s preceded by roughly 25 s rest and 5 s baseline recording. The task was either to increase (“activation”) or decrease prefrontal O_2_Hb concentration (“deactivation”) whereby up- and down-regulation trials were equally likely and randomly serialized^32^.

Contrary to the original plan, in the software we acquired from the company O_2_Hb amplitudes were inverted for polarity. Positive SCP shifts are associated with deactivation and negative shifts are associated with activation. However, positive O_2_Hb amplitudes reflect activation and negative O_2_Hb amplitudes reflect deactivation. Unfortunately, this was not considered in the software and the error remained unnoticed until the analysis of the data. As participants were informed that positive deflections reflected cortical “activation”, the “trial and error” situation during strategy testing may have been counterintuitive for them. Moreover, such an inverted polarity bears the risk of compromising transfer into daily life as strategies do not match the intended behavior.

We consulted the Psychiatry Department’s representative of the Ethics Committee for the Medical Department, University of Tübingen to decide how to proceed with this problem and whether patients should be informed. He recommended not informing patients in the fNIRS group if symptom data showed that the inverted polarity did not cause disadvantage for them. To clarify whether we did any harm to the patients we conducted the following analysis the results of which are reported in the results section:

(1) Reliable Change (RC) Index^41^: $RC = \frac{{(x_{1 - } x_{2} )}}{{\sqrt {2*(SD_{1} *\sqrt {1 - r_{tt} } )^{2}} }}$ (where *x*_1_ and *x*_2_ specify baseline- and post test score; *SD*_1_ specifies the standard deviation of baseline observations and, *r*_*tt*_ specifies reliability of the measure).

and (2) Clinically Significant Change giving the percentage of improved, unchanged and deteriorated cases in all feedback groups to show that the corrupt fNIRS protocol did not lead to a higher rate of deterioration compared to the other conditions.

#### Electromyogram biofeedback

For the EMG-BF, Ag/AgCl ring electrodes were placed over the right and left supraspinatus muscles and EEG electrodes were attached analogously to the SCP-NF. The relation between relaxation on the left and tension of the right muscle was used as the feedback signal when participants were asked to regulate the signal up (vice versa for downregulation)^32^. Trial length, visual output, transfer trials and overall duration were the same as in the SCP-NF. Participants in the EMG training were not aware of their randomization to a semi-active control condition*.*

#### Clinical and cognitive outcome measures

Primary outcome, i.e. changes in core symptoms, comprised an ADHD self-rating questionnaire (ADHS-SB^36^), the clinician-rated WRI^36^ as well as the German third-party questionnaire FEA-AFB designed to evaluate current symptoms of ADHD in adulthood^37^. Our choice of multiple measures to assess the primary outcome to picture symptom changes allow representative statements about the efficacy of neurofeedback in adult patients with ADHD across different sources of information. Secondary outcomes included cognitive factors, namely attention (d2-R^42^) and intelligence (CFT-20-R^31^). Furthermore, changes in specific ERP- and fNIRS parameters related to cognitive preparation, attention, response inhibition and working memory as well as self-regulation performance^32^ were among the secondary outcome variables. In every fifth NF session, participants completed a questionnaire (FERT^43^) to assess non-specific effects of feedback training (expectation, fit between therapist and patient, therapeutic relationship, therapist expertise, persuasiveness of the therapist, willingness of the patient to engage) on a 7-point Likert scale. The sample size calculation was based on a power calculation of a meta-analysis by Arns et al.^44^ and has been described in detail in the published study protocol^32^.

#### ERP outcome measures

We used the NeXus-32 DC amplifier (Mind Media B.V. with Biotrace + Software, Herten, Netherlands) for electrophysiological recordings. We placed 20 EEG electrodes in a cap in accordance with the international 10–20 montage system, referenced against mastoid A2 with a ground electrode on mastoid A1. EEG data were recorded with a sampling rate of 512 Hz. We attached two vertical and two horizontal pregelled Ag/AgCl electrodes to record eye movements and blinks. DC offset was kept below 25 000 μV peak-to-peak^32^. The EEG assessments lasted about two hours including preparation time and comprised resting state EEG (15 min eyes closed, 5 min eyes open) and three active paradigms (Go/NoGo CNV task, P300 acoustic counting task and P300 reaction time task). The sound pressure level of all tones in all tasks was 90 dB presented via two speakers placed at a distance of 1 m from the participant with a 0.5 m horizontal distance from each other. All instructions were presented with a recorded female voice. Participants were seated in a comfortable EEG investigation chair during the recording^45^.

#### Go/NoGo task (contingent negative variation, CNV)

A warning stimulus (500 Hz; 50 ms; n = 200) preceded a second stimulus which could be a NoGo low-pitched (1000 Hz; 50 ms; n = 150) or a Go high-pitched tone (2000 Hz; 50 ms; n = 50). The subjects were instructed to keep their eyes closed and press the spacebar of a computer keyboard with their dominant hand as quickly as possible whenever the Go-tone was presented. The time span between the warning stimulus and the second stimulus was constantly 1.8 s, whereas the time between trials varied randomly between 2.0 and 2.4 s. The task duration was 13 min^45^.

#### P300 acoustic counting task

The auditory stimuli were presented for 50 ms in pseudo-randomized order with a delay of 1300 ms. The participants were instructed to mentally count the rare high target tones (1500 Hz; n = 49) between more frequent distractor stimuli (1000 Hz; n = 351) keeping their eyes closed. Target sounds appeared with a probability of 12.25%. The task lasted 10 min^45^.

#### fNIRS outcome measures

The fNIRS assessments were conducted in a separate session comprising a Go/NoGo task, an n-back task and a verbal fluency task in randomized order. The whole session lasted about 60 min, including preparation time. Participants were seated in a comfortable chair in front of a monitor at a distance of approximately 80 cm in a completely dark and sound-attenuated room. Standardized instructions were given by the investigator. For the Go/NoGo- and n-back tasks, instructions were additionally presented on the computer screen. Before the start, a baseline was determined over 10 s. The conditions were implemented in an alternate fashion. The 30-s task blocks were separated by 30-s periods of rest during which participants were asked to sit still and relax.

#### n-back task

Participants were presented with a flow of white letters against a black background on a computer screen in pseudorandom sequence (300 ms; interstimulus interval 1700 ms). Participants were instructed to press the spacebar of a standard computer keyboard as fast as possible either whenever the displayed letter was identical to the penultimate one (2-back condition; high working memory load) or to the preceding letter (1-back condition; low working memory load), or whenever the letter ‘O’ was displayed on the screen (0-back condition; control condition). All conditions were repeated three times, so that participants completed a total of 9 task blocks. For all three conditions, 12 target trials appeared across task blocks and the task lasted 9 min^32,46^.

#### Go/NoGo task

Participants were presented with a flow of white letters presented in pseudo-random sequence (500 ms; interstimulus interval 1500 ms) against a black background. During Go blocks, subjects were prompted to press the spacebar of a computer keyboard as fast as possible whenever any letter appeared on the screen. During NoGo blocks, participants were asked to respond to any letter on the screen but to inhibit the motor response when the presented letter was an “N”. Both conditions were repeated four times, so that participants completed a total of 8 task segments. 8 targets and 8 distractors were presented per NoGo block and the task lasted 8 min. In the Go condition, no “N” was presented^32,46^.

### Statistical analysis and preprocessing

We used IBM SPSS Statistics version 26.0 (Armonk, NY, USA) and Microsoft Excel 2014 for statistical analyses. We analyzed clinical, behavioral and neurocognitive as well as EEG- and fNIRS data with mixed ANOVAs including group- and assessment factors (three groups × four assessment points; cf.^32^; for learners vs. non-learners: five groups × four assessment points) followed by Bonferroni-corrected post-hoc tests. Where appropriate, Tukey HSD post-hoc tests were used to assess group differences. For some data, normality or variance homogeneity assumptions were not fulfilled. However, we will report ANOVA results as with comparable sample sizes, ANOVA is quite robust. To evaluate the magnitude of treatment effects, Reliable Change (RC) Index and Clinically Significant Change were calculated. For all analyses, the significance level was set to *p* < .05 and Bonferroni-corrected. We report 2-tailed probabilities and effect sizes (ANOVA: *η*_*p*_^2^; paired *t*-test: Cohen’s *d*, Kruskal–Wallis test*:* Cohen’s *d*, Wilcoxon signed-rank test: *r*; Welch ANOVA: ω^2^, Tukey HSD: g*). Missing data (< 5%) were assumed to be random and were replaced using the EM algorithm implemented in SPSS. Details on pre-processing and statistical analysis of EEG- as well as fNIRS data can be found in the supplementary material.

#### Learners versus non-learners

We categorized learners and non-learners according to earlier considerations that the ability to differentiate between upregulation and downregulation during the transfer condition is the highest and most important level of self-regulation that can be achieved (cf.,^45^). Furthermore, this approach considers the fairly frequently observed initial success that, however, occurs rather by chance and is most often followed by a temporary decrease in regulation performance. Using a pre-post comparison or linear increase as a criterion, these data might lead to the conclusion that participants did not improve their regulation skills. We used the mean of two sessions (28 and 29) in the active groups to prevent that artifacts or single diverging sessions distort the outcome.

### Human subjects informed consent

Prior to inclusion, written informed consent after receiving detailed information about the study procedure was obtained from the patients. The study was reviewed and approved by the ethics committee of the University of Tübingen, and all procedures involved were in accordance with the latest version of the Declaration of Helsinki.

## Results

### Sample characteristics

Apart from a significant difference between participants in SCP- and EMG group in WURS-K scores, there were no group differences at baseline in demographic, clinical or medication status factors (Table 1).

### Slow cortical potential neurofeedback

**Figure 3:**
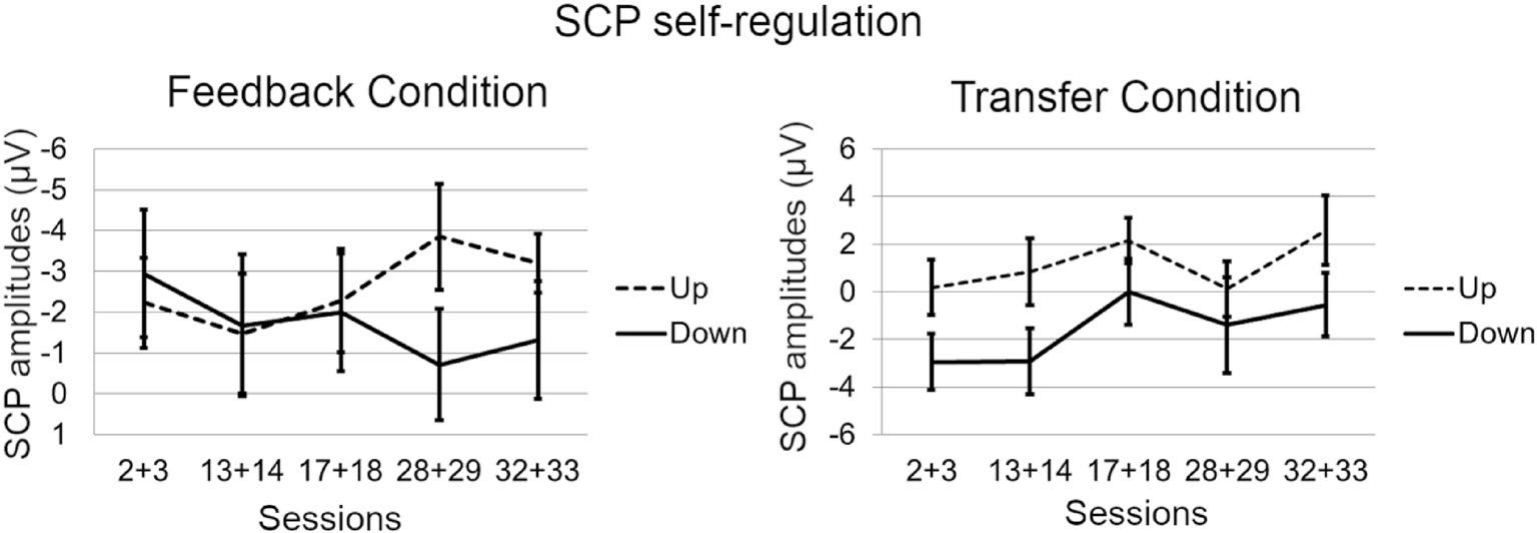
Self-regulation of SCP amplitude by task (polarity; upregulation vs. downregulation) and condition (feedback vs. transfer).

Repeated measures ANOVA did not reveal a significant main effect of “session” for SCP amplitudes in any of the tasks or conditions, although the direction of the amplitudes over the training course went into the desired direction (Fig. 3).

### Functional near-infrared spectroscopy neurofeedback

**Figure 4:**
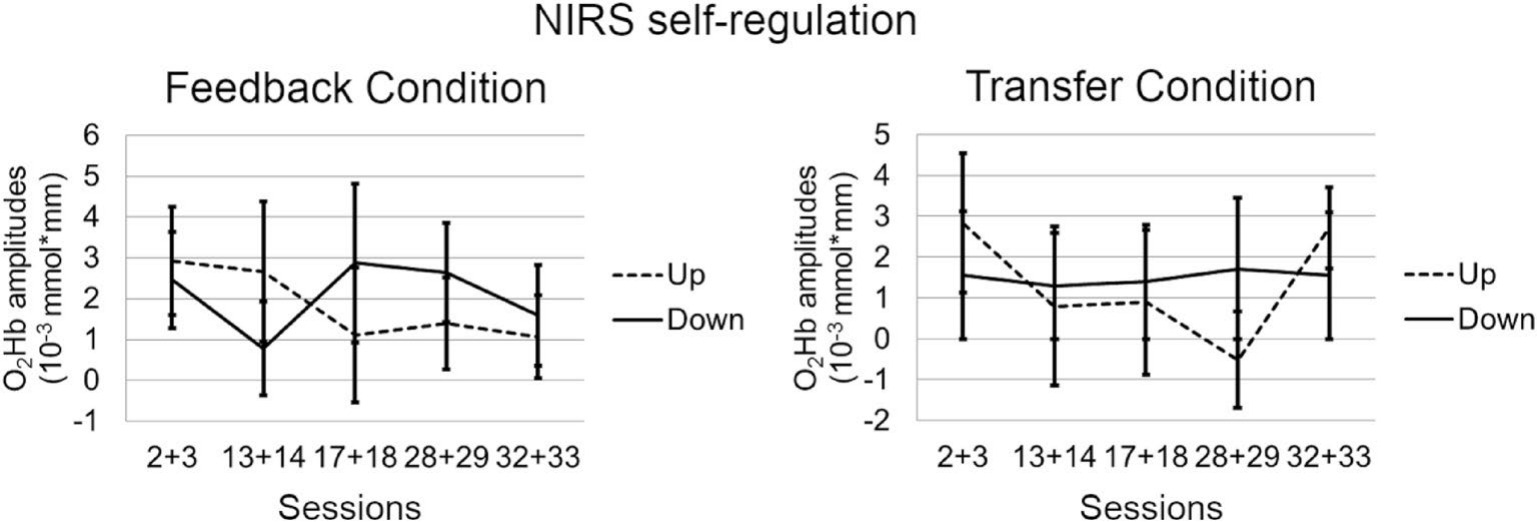
Self-regulation of O_2_Hb amplitude by task (polarity; upregulation vs. downregulation) and condition (feedback vs. transfer) averaged over left and right ROI channels. The figure shows the non-inverted O_2_Hb amplitudes (opposite to the online feedback signal).

Repeated measures ANOVA did not reveal a significant main effect of “session” for O_2_Hb amplitudes in any of the tasks or conditions in the fNIRS group. There was no consistent pattern of the hemodynamic response in up- versus downregulation (Fig. 4).

### Learners versus non-learners

According to the differentiation of up- versus downregulation in the transfer condition during sessions 28 and 29, 8 (30.8%) participants in the SCP group and 13 (61.9%) participants in the fNIRS group were classified as learners. In the SCP group, three of the learners took medication (50% of medicated subjects in the SCP group); in the fNIRS group, four of the learners were on medication (57.1% of medicated subjects in the fNIRS group).

Regarding non-specific effects of the training, we did not observe any significant group differences between participants in the SCP-, fNIRS or EMG training in expectation, fit between therapist and patient, therapeutic relationship, persuasiveness of the therapist or willingness of the patient to engage over the training course (descriptive statistics in Table S1 in the supplementary material). We did not observe any significant differences on either of the factors when looking at learners and non-learners separately. However, we found a significant increase regarding perceived therapist expertise over the training course in all groups (*Greenhouse–Geisser* *F*(3.89, 248.91) = 4.46, *p* = .002, *η*_*p*_^2^ = 0.065; significant increase between session 5 and 10 (1.13, *p* = .036)). When separating learners and non-learners we observed an additional main effect of group (*F*(4,62) = 2.77, *p* = 0.035, *η*_*p*_^2^ = 0.151) with fNIRS non-learners perceiving the therapist less competent as compared to fNIRS learners (Session 5: *t*(19) =  − 2.20, *p* = .025 *d*_Cohen_ = − 0.991; Session 10: *t*(19) = − 2.74, *p* = .013, *d*_Cohen_ = − 1.232; Session 15: *t*(19) = − 3.48, *p* = .003, *d*_Cohen_ = − 1.563; Session 20: *t*(19) = − 3.18, *p* = 0.011, *d*_Cohen_ = − 1.657**;** Fig. S1 in the Supplements).

### Clinical and cognitive outcome measures

**Figure 5:**
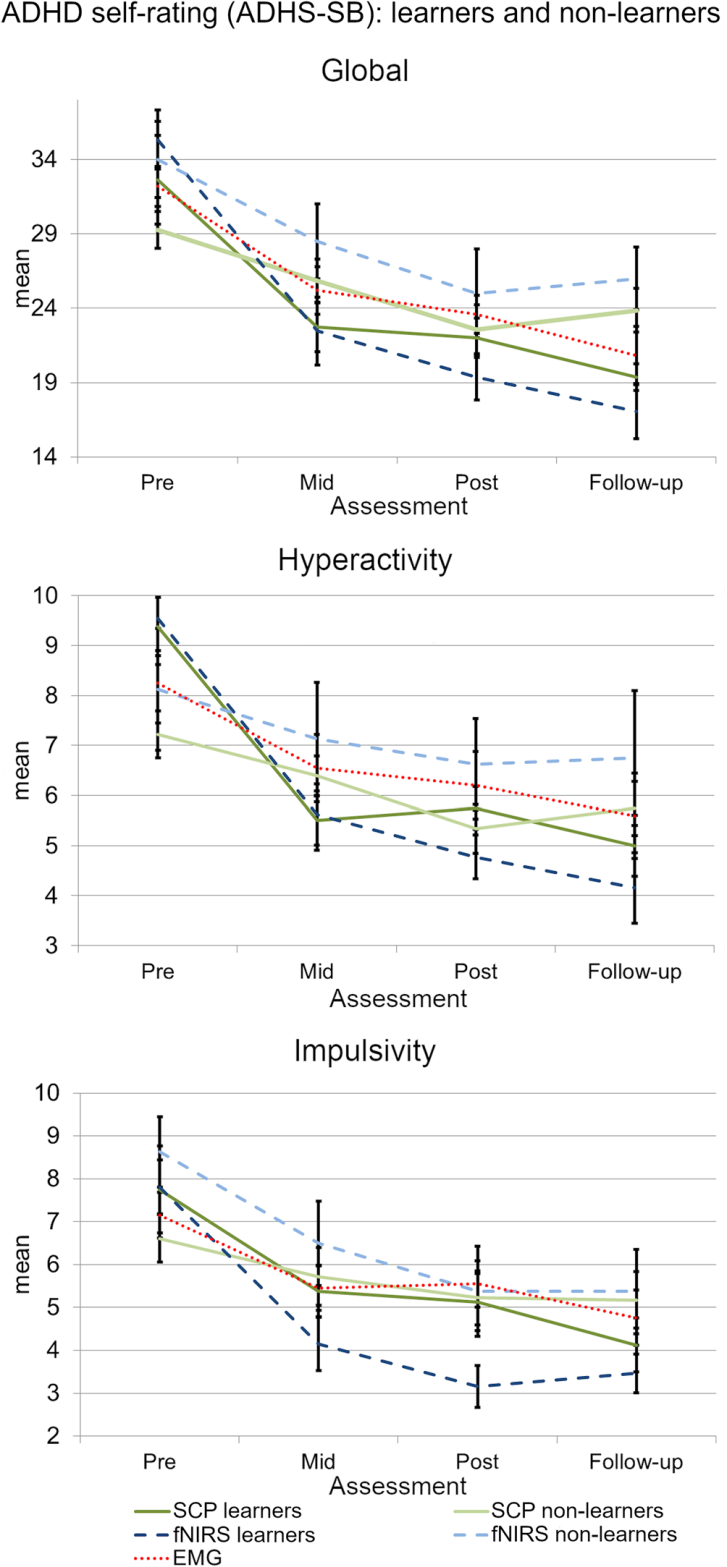
ADHD self-rating differentiating learners from non-learners in fNIRS- and SCP-NF compared to EMG-BF.

Analyses of the longitudinal course across assessments (information on mean and standard deviation see Table S2 in the Supplements) from pre-test to 6-months FU provided statistically significant improvement on the ADHS-SB global score for all groups (*p* < .001) with no group-by-time interaction. Six paired samples post-hoc tests (Bonferroni-adjusted significance level α = 0.008) indicate significant reductions in the ADHS-SB global score between pre- and mid-, pre- and post-, mid- and post- as well as pre-training and FU. The inattention-, hyperactivity- as well as impulsivity subscales also showed a statistically significant decrease over time (Table 2), again without a group-by-time interaction or main effect for group. Six post-hoc paired samples tests, here again, indicate significant symptom reductions between pre- and mid-, pre- and post-, as well as pre-training and FU for all three subscales (Table 3). When looking at learners and non-learners separately, mixed ANOVAs revealed a “learn group” by time interaction on the global scale (*Greenhouse–Geisser* *F*(10.04,155.62) = 2.35, *p* = .013, *η*_*p*_^2^ = 0.131) as well as on the hyperactivity- (*Greenhouse–Geisser* *F*(9.83,152.29) = 1.94, *p* = .032, *η*_*p*_^2^ = 0.111) and impulsivity subscales (*F*(2.71,167.81) = 2.91, *p* = .043, *η*_*p*_^2^ = 0.107). Furthermore, there was a main effect of time on the inattention subscale (*F*(12,186) = 34.32, *p* < .001, *η*_*p*_^2^ = 0.356) with significant symptom reduction between pre- and mid- (3.33, *p* < 0.001), pre- and post- (4.72, *p* < .001) as well as pre-assessment and FU (5.20, *p* < 0.001). Post-hoc tests further revealed that on the global scale the learn groups differed significantly at FU (*F*(4,26.59) = 3.55, *p* = .019, *ω*^2^ = 0.132) with SCP non-learners (6.79, *p* = .081, g* = 1.021) as well as fNIRS non-learners (8.92, *p* = .054, g* = 1.322) providing statistically non-significantly higher scores compared to fNIRS learners (Fig. 5). For further analysis of the interaction effect, we also tested whether the difference between FU and pre-treatment assessment differed between learners and non-learners in the respective training conditions. Kruskal–Wallis test showed that there was a significant difference of global symptom change (*H* = 13.28, *p* = .010). Pairwise comparisons showed significantly more pronounced symptom changes in fNIRS learners compared to EEG non-learners (*p* = .005). On the hyperactivity subscale, post-hoc tests or Kruskal–Wallis test did not reveal any significant effects. The learn groups differed, however, in the impulsivity self-ratings at post-treatment assessment (*F*(4,24.13) = 3.02, *p* = .038, *ω*^2^ = 0.108) with the EMG group providing statistically non-significantly higher scores compared to the fNIRS learners (2.40, *p* = 0.065, g* = 0.943).

**Table 2:** Symptom ratings before feedback training, after half of the sessions and after feedback training for each group.

	Time effect	Treatment effect	Interaction
	Test statistic; effect size	Test statistic; effect size	Test statistic; effect size
^§^ADHS-SB Global	^+^*F*(2.45,156.88) = 54.84; ***p* < .001**; *η*_*p*_^2^ = 0.460	*F*(2,64) = 0.05; *n.s*.; *η*_*p*_^2^ = 0.002	^+^*F*(4.88,151.42) = 1.79; *n.s*; *η*_*p*_^2^ = 0.050
^§^ADHS-SB Inattention	^+^*F*(2.66,170.15,) = 40.97; ***p* < .001**; *η*_*p*_^2^ = 0.390	*F*(2,64) = 0.13; *n.s.*; *η*_*p*_^2^ = 0.004	^+^*F*(5.32,170.15) = 1.43; *n.s*; *η*_*p*_^2^ = 0.043
^§^ADHS-SB Hyperactivity	^+^*F*(2.43,155.38) = 26.23 ***p* < .001**; *η*_*p*_^2^ = 0.291	*F*(2,64) = 0.22; *n.s.*; *η*_*p*_^2^ = 0.007	^+^*F*(4.86,155.38) = 0.72; *n.s*; *η*_*p*_^2^ = 0.022
^§^ADHS-SB Impulsivity	*F*(3,192) = 34.39; ***p* < .001**; *η*_*p*_^2^ = 0.349	*F*(2,64) = 0.21; *n.s.*; *η*_*p*_^2^ = 0.074	*F*(6,192) = 2.91; *n.s*; *η*_*p*_^2^ = 0.074
^§^WRI Global	^+^*F*(1.82,116.35) = 31.53; ***p* < .001**; *η*_*p*_^2^ = 0.330	*F*(2,64) = 1.01; *n.s*.; *η*_*p*_^2^ = 0.031	^+^*F*(3.64,116.35) = 0.08; *n.s.;* *η*_*p*_^2^ = 0.002
^§^WRI Inattention	*F*(2,128) = 22.44; ***p* < .001**; *η*_*p*_^2^ = 0.260	*F*(2,64) = 0.05; *n.s*.; *η*_*p*_^2^ = 0 .001	*F*(4,128) = 0.79; *n.s*.; *η*_*p*_^2^ = 0.024
^§^WRI Hyperactivity	*F*(2,128) = 12.08; ***p* < .001**; *η*_*p*_^2^ = 0.159	*F*(2,64) = 0.86; *n.s*.; *η*_*p*_^2^ = 0.026	*F*(4,128) = 0.88; *n.s*.; *η*_*p*_^2^ = 0.027
^§^WRI Impulsivity	*F*(2,128) = 11.33; ***p* < .001**; *η*_*p*_^2^ = 0.150	*F*(2,64) = 0.24; *n.s*.; *η*_*p*1*.*01_^2^ = 0.007	*F*(4,128) = 1.10; *n.s*.; *η*_*p*_^2^ = 0.031
*FEA current	^+^*F*(2.42,154.56) = 16.47; ***p* < .001**; *η*_*p*_^2^ = 0.205	*F*(2,64) = 0.07; *n.s*.; *η*_*p*_^2^ = 0.002	^+^*F*(4.83,154.56) = 0.43; *n.s*.; *η*_*p*_^2^ = 0.013
^§^BDI	^+^*F*(2.39,152.92) = 19.85; ***p* < .001**; *η*_*p*_^2^ = 0.237	*F*(2,64) = 0.16; *n.s*.; *η*_*p*_^2^ = 0.005	^+^*F*(4.78,152.92) = 0.54; *n.s.;* *η*_*p*_^2^ = 0.017

*Note.* *ADHS-SB* = German ADHD self-rating scale for symptoms in adulthood^35,36^; *BDI* = Beck-Depression Inventory^47^; *EMG* = Electromyography; *d*_Cohen_ = , effect size Cohen’s d; *FU* = Follow-up; *n.s*. = not significant; *SCP* = Slow Cortical Potential; *SD* = Standard Deviation; *WRI* = Wender-Reimherr-Interview^35,36^: assessment at pre-, post- and follow-up assessment.

^+^Greenhouse–Geisser corrected.

^§^Information missing for 2 participants at follow-up.

*Information missing for 15 participants at pre-, for 32 participants (46.3%) at mid-, for 37 participants (53.7%) at post-training and for 43 participants (62.7%) at follow-up.

**Table 3:** Post-hoc analyses of symptom ratings before feedback training, after half of the sessions, after feedback training and at 6-months follow-up for each group.

	Time effect			
	Test statistic; effect size			
	pre versus mid	pre versus post	mid versus post	pre versus FU
ADHS-SB Global	*t*(66) = 8.89; *p* < .001; *d*_Cohen_ = 1.086	*t*(66) = 10.73; *p* < .001; *d*_Cohen_ = 1.311	*t*(66) = 2.82; *p* = .006; *d*_Cohen_ = 0.345	*t*(66) = 8.85; *p* < .001; *d*_Cohen_ = 1.081
ADHS-SB Inattention	*t*(66) = 7.07; *p* < .001; *d*_Cohen_ = 0.864	*t*(66) = 9.26; *p* < .001; *d*_Cohen_ = 1.131	*n.s*	*t*(66) = 8.14; *p* < .001; *d*_Cohen_ = 0.994
ADHS-SB Hyperactivity	t(66) = 6.12; *p* < .001; *d*_Cohen_ = 0.611	*Z* = -5.83; *p* < .001; *r* = 0.712	*n.s*	*Z* = − 5.23; *p* < .001; *r* = 0.639
ADHS-SB Impulsivity	*Z* = − 5.32; *p* < .001; *r* = 0.650	*Z* = − 5.92; *p* < .001; *r* = 0.723	*n.s*	*Z* = − 5.70; *p* < .001; *r* = 0.696
WRI Global	–	*t*(66) = 7.04; *p* < .001; *d*_Cohen_ = 0.860	–	*t*(66) = 6.24; *p* < .001; *d*_Cohen_ = 0.762
WRI Inattention	–	*Z* = − 4.54; *p* < .001; *r* = 0.556	–	*Z* = − 5.18; *p* < .001; *r* = 0.632
WRI Hyperactivity	–	*Z* = − 4.24; *p* < .001; *r* = 0.518	–	*Z* = − 3.14; *p* = .002; *r* = 0.384
WRI Impulsivity	–	*Z* = − 4.26; *p* < .001; *r* = 0.520	–	*Z* = − 3.06; *p* < .001; *r* = 0.374
BDI	*Z* = − 5.53; *p* < .001; *r* = 0.676	*Z* = − 5.08; *p* < .001; *r* = 0.621	*n.s*	*Z* = − 4.29; *p* < .001; *r* = 0.524

*Note:* Only significant post-hoc results are reported. *ADHS-SB* = German ADHD self-rating scale for symptoms in adulthood^35,36^; *BDI* = Beck-Depression Inventory^47^; *EMG* = Electromyography; *d*_Cohen_ = effect size Cohen’s d; *FU* = Follow-up; *n.s*. = not significant; *SCP* = Slow Cortical Potential; *SD* = Standard Deviation; *WRI* = Wender-Reimherr-Interview^35,36^: assessment at pre-, post- and follow-up assessment.

**Figure 6:**
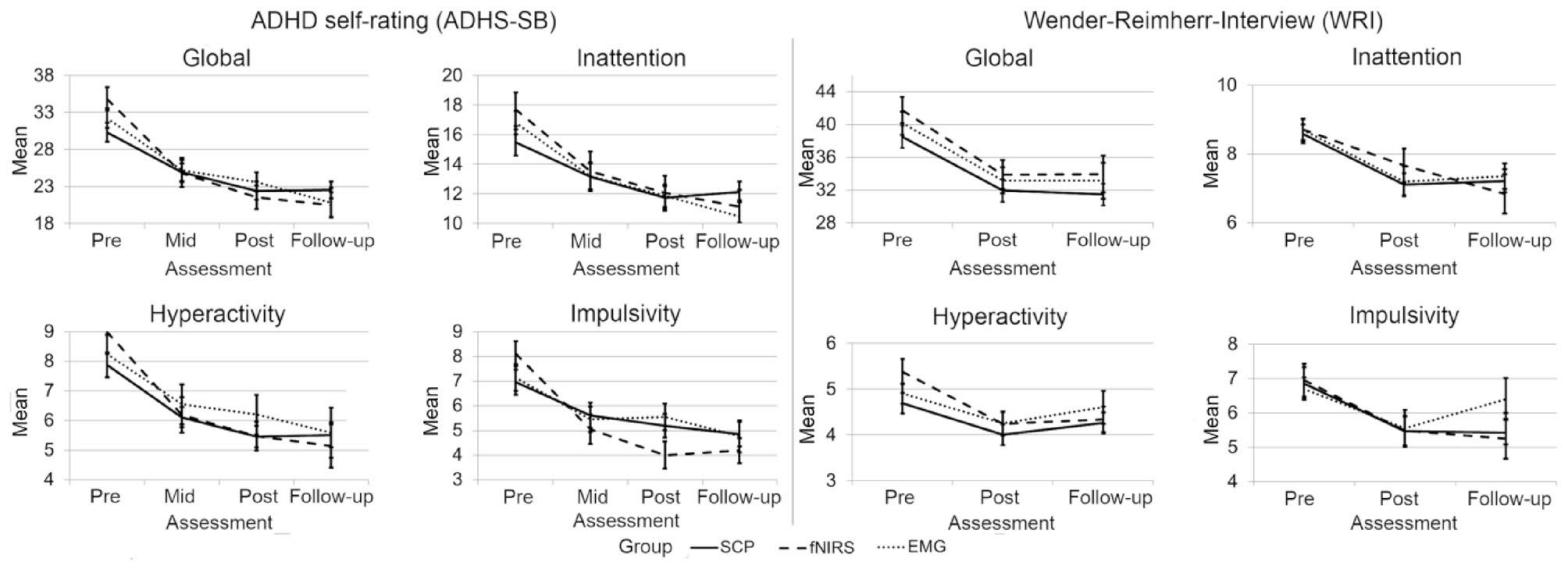
Clinical trajectories for primary outcome assessments ADHS-SB and WRI on the global scale as well as on inattention-, hyperactivity- and impulsivity subscales.

Analyses of the assessments from pre-test to 6-months FU provided a statistically significant decline of the WRI global score as well as a reduction of symptoms on the inattention-, hyperactivity- and impulsivity subscales for all groups (*p* < .001) with no group-by-time interaction or main effect for group. Three paired samples post-hoc tests (Bonferroni-adjusted significance level α = 0.017) indicate a significant difference between pre- and post-training as well as pre-training and FU (Table 3). When looking at learners and non-learners separately, mixed ANOVA revealed no additional significant effects for neither learn group. Figure 6 shows the clinical trajectories for primary outcome assessments ADHS-SB and WRI.

Analyses of the assessments from pre-test to 6-months FU provided a statistically significant decline of the FEA across groups but no group-by-time interaction or main effect for group (Table 2). Post-hoc tests did not reveal any statistically significant effects after correction for multiple comparisons.

BDI scores are depicted in Fig. S2. Similar results as in the primary outcome were observed with a significant main effect of time without significant group-by-time interaction (Table 2). Six post-hoc paired samples tests, here again, indicate a significant symptom reduction between pre- and mid-training, pre- and post-training, as well as pre-training and FU (Table 3).

Mixed ANOVA from pre-test to 6-months FU provided statistically significant improvement on the d2-R for all groups (*F*(2,128) = 45.19, *p* < .001, *η*_*p*_^2^ = 0.414 for concentration; *Greenhouse–Geisser* *F*(1.76,112.41) = 21.96, *p* < .001, *η*_*p*_^2^ = 0.255 for operation speed; *Greenhouse–Geisser* *F*(1.81,115.51) = 17.41, *p* < .001, *η*_*p*_^2^ = 0.214 for accuracy; information on mean and standard deviation see Table S3 in the Supplements) but no group- or group-by-time interaction effects. Three post-hoc Wilcoxon tests (Bonferroni-adjusted significance level α = 0.017) indicate a significant difference between pre- and post-training (*Z* = − 5.58, *p* < .001, *r* = 0.682 for concentration; *t*(66) =  − 4.28, *p* < .001, *d* = 0.522 for operation speed; *Z* =  − 4.19, *p* < .001, *r* = 0.512 for accuracy), post-training and FU (*Z* =  − 3.42, *p* = .001, *r* = 0.418 for concentration; *Z* =  − 3.32, *p* = .001, *r* = 0.406 for operation speed) as well as pre-training and FU (*Z* =  − 6.00, *p* < .001, *r* = 0.733 for concentration; *Z* =  − 4.95, *p* < .001, *r* = 0.605 for operation speed; *Z* =  − 5.05, *p* < .001, *r* = 0.617 for accuracy).

Analysis of the time course from pre- to post-test provided statistically significant improvement on the CFT-20-R for all groups with no group-by-time interaction or group effects (*Greenhouse–Geisser* *F*(1.46, 93.71) = 24.95, *p* < .001, *η*_*p*_^2^ = 0.280; information on mean and standard deviation see Table S3 in the Supplements).

The RCI was computed for each measure using the available reliability coefficients in the respective primary outcome test manuals (ADHS-SB and WRI) and the standard deviation of the sample at pre-assessment. Table 4 shows the proportion of patients categorized as undergoing a clinically important improvement or deterioration. Fisher-Freeman-Halton test revealed a significantly higher proportion of clinically significantly improved (*p* < .001) as well as improved (*p* < .001) impulsivity ratings at post-assessment in the NIRS group. The proportions of patients in the training groups in all other scores were similar.

**Table 4:** Categorization of clinically significant change.

	POST								
	SCP (*n* = 26)			fNIRS (*n* = 21)			EMG (*n* = 20)		
	Improvement	No Change	Deterioration	Improvement	No Change	Deterioration	Improvement	No Change	Deterioration
ADHS-SB Subscale	% (*n*)			% (*n*)			% (*n*)		
Global	53.85 (14)	46.15 (12)	0 (0)	71.43 (15)	28.57 (6)	0 (0)	70.00(14)	30.00 (6)	0 (0)
Inattention	30.77 (8)	69.23 (18)	0 (0)	57.14 (12)	42.86 (9)	0 (0)	55.00 (11)	45.00 (9)	0 (0)
Hyperactivity	30.77 (8)	69.23 (18)	0 (0)	23.81 (5)	76.19 (16)	0 (0)	15.00 (3)	85.00 (17)	0 (0)
Impulsivity	19.23 (5)	80.77 (21)	0 (0)	71.43(15)	28.57 (6)	0 (0)	20.00 (4)	80.00 (16)	0 (0)
WRI subscale									
Global	38.46 (10)	61.54 (16)	0 (0)	38.10 (8)	61.90 (13)	0 (0)	35.00 (7)	65.00 (13)	0 (0)
Inattention	26.92 (7)	69.21 (18)	3.84 (1)	23.81 (5)	71.43 (15)	4.76 (1)	35.00 (7)	60.00 (12)	5.00 (1)
Hyperactivity	7.69 (2)	92.31 (24)	0 (0)	19.05 (4)	80.95 (17)	0 (0)	20.00 (4)	75.00 (15)	5.00 (1)
Impulsivity	19.23 (5)	80.77 (21)	0 (0)	19.05 (4)	80.95 (17)	0 (0)	30.00 (6)	65.00 (13)	5.00 (1)

	FOLLOW-UP								
	SCP (*n* = 25)			fNIRS (*n* = 21)			EMG (*n* = 20)		
	Improvement	No Change	Deterioration	Improvement	No Change	Deterioration	Improvement	No Change	Deterioration
ADHS-SB Subscale	% (*n*)			% (*n*)			% (*n*)		
Global	64.00 (16)	32.00 (8)	4.00 (1)	76.19 (16)	23.81(5)	0 (0)	^+^63.16 (12)	^+^36.84 (7)	^+^0 (0)
Inattention	40.00 (10)	56.00(14)	4.00 (1)	57.14 (12)	42.85 (9)	0 (0)	^+^57.89 (11)	^+^42.11 (8)	^+^0 (0)
Hyperactivity	32.00 (8)	64.00 (16)	4.00 (1)	23.81 (5)	76.19 (16)	0 (0)	^+^26.32 (5)	^+^68.42 (13)	^+^5.26 (1)
Impulsivity	28.00 (7)	68.00(17)	4.00 (1)	61.90 (13)	38.10 (8)	0 (0)	^+^26.32 (5)	^+^73.68 (14)	^+^0 (0)
WRI subscale									
Global	^+^34.78 (8)	^+^65.22 (15)	^+^0 (0)	31.58(6)	68.42 (13)	0 (0)	50.00 (10)	45.00 (9)	5.00 (1)
Inattention	^+^30.43 (7)	^+^69.57 (16)	^+^0 (0)	36.84 (7)	63.16 (12)	0 (0)	25.00 (5)	70.00 (14)	5.00 (1)
Hyperactivity	^+^13.04 (3)	^+^82.61(19)	^+^4.35 (1)	10.52 (2)	89.48 (17)	0 (0)	15.00 (3)	75.00 (15)	10.00 (2)
Impulsivity	^+^13.04 (3)	^+^86.96 (20)	^+^ 0 (0)	26.32 (5)	73.68 (14)	0 (0)	15.00 (3)	70.00 (14)	15.00 (3)

*Note:* Reliable Change Index (RCI) was based on change from pre- to post test and from pre test to follow-up. Reliable improvement is defined as RCI ≤ 1.96, Reliable deterioration is defined as RCI ≥ 1.96. *ADHS-SB* = German ADHD self-rating scale for symptoms in adulthood^35,36^; *WRI* = Wender-Reimherr-Interview^35,36^.

^+^Information missing for 1 participant.

### ERP outcome measures

#### Go/NoGo task (CNV)

**Figure 7:**
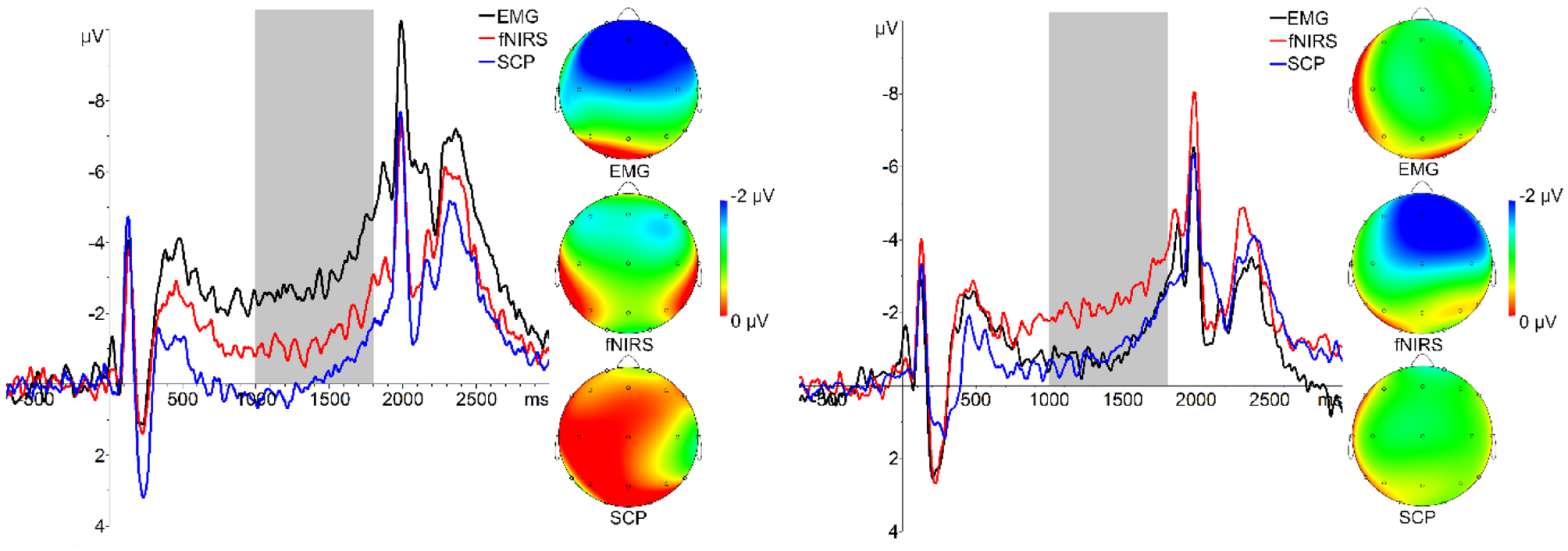
ERP plots at Fz and scalp topographies at time window 1000–1800 ms depicting contingent negative variation (CNV) at pre- and post-assessment in the SCP-, fNIRS- and EMG group. The shaded area indicates the time window in which CNV was measured.

Information on mean, standard deviation and range of performance are listed in the Supplements (Table S4). Mixed ANOVA revealed a significant reduction of reaction times over the training in all groups (*Greenhouse–Geisser* *F*(2.09,135.83) = 10.11, *p* < .001, *η*_*p*_^2^ = 0.135). Six paired samples post-hoc tests (Bonferroni-adjusted significance level α = 0.008) show a significant reduction of the reaction times between pre- and mid-training (*Z* = − 2.98, *p* = .003, *r* = 0.364), pre- and post-training (*Z* =  − 3.49, *p* < .001, *r* = 0.426) as well as pre-training and FU (*Z* =  − 4.73, *p* < .001, *r* = 0.578). Mixed ANOVA revealed a significant group-by-time interaction effect for CNV amplitudes (*Greenhouse–Geisser* *F*(4.83,154.61) = 4.82, *p* < .001, *η*_*p*_^2^ = 0.131). Post-hoc Welch-test identified significant differences at baseline (*F*(2,36.74) = 5.63, *p* = 0.001, *ω*^2^ = 0.121) and Tukey HSD revealed that the statistically significant difference was between the fNIRS- and the EMG group (2.02, *p* = 0.045, g* = 0.724) as well as between the SCP- and the EMG group (3.17, *p* < 0.001, g* = 1.035; Fig. 7) with the EMG group showing more negative amplitudes which vanishes over the training course.

#### P300 acoustic counting task

**Figure 8:**
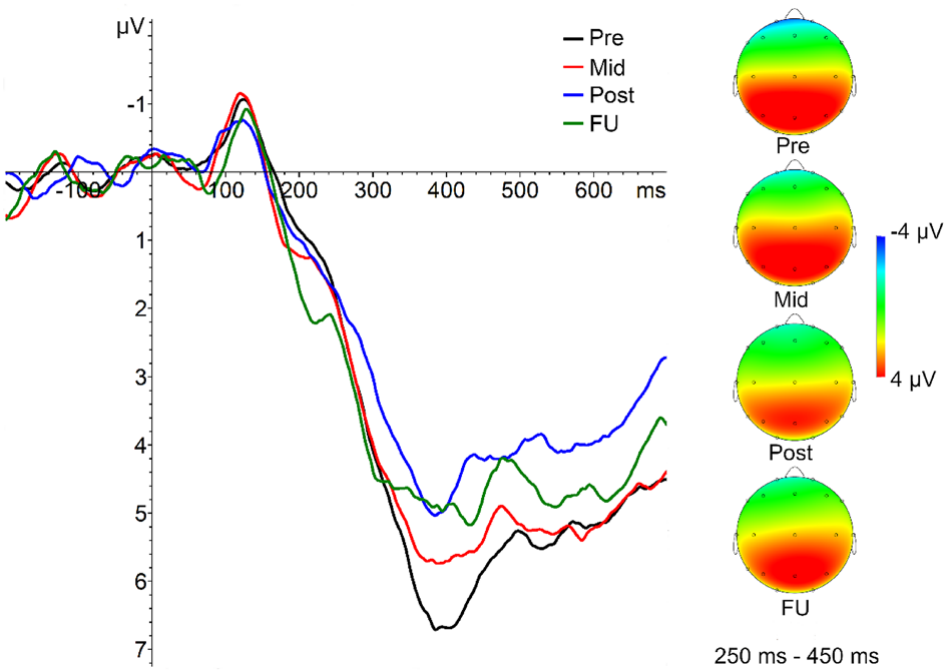
P300 amplitudes averaged over all feedback groups at pre-, mid-, post- and follow-up assessment at Pz and scalp topographies at time window 250–450 ms.

Mean, standard deviation and range of counting errors are presented in the Supplements (Table S5). Mixed ANOVA revealed no significant effects on performance level. Analysis of electrophysiological data revealed a significant reduction in P300 amplitude over the training in all groups (*Greenhouse–Geisser* *F*(2.63,168.47) = 5.67, *p* = .002, *η*_*p*_^2^ = 0.081) with no group or group-by-treatment interaction effects (Fig. 8). Six paired samples post-hoc tests (Bonferroni-adjusted significance level α = 0.008) reveal that this reflects a significant reduction of the P300 between pre- and post-training (*t*(66) = 3.75, *p* < .001, *d*_Cohen_ = 0.458) as well as mid- and post-training (*t*(66) = 3.11, *p* = .003, *d*_Cohen_ = 0.380).

### fNIRS outcome measures

#### n-back task

Information on mean, standard deviation and range of behavioral data are available in the Supplements (Table S6). There was a significant main effect for overall reaction times over the training course (*F*(3,192) = 7.79, *p* < .001, *η*_*p*_^2^ = 0.109). Overall reaction times significantly declined between pre- and mid-training (*Z* =  − 3.12, *p* = .002, *r* = 0.381), pre-training and FU (*Z* =  − 3.80, *p* < .001, *r* = 0.464) as well as between post-training and FU (*t*(66) =  − 3.24, *p* = .002, *d*_Cohen_ = 0.396). Mixed ANOVA revealed the same pattern for reaction times in the 1-back- (*F*(3,192) = 6.03, *p* = .001, *η*_*p*_^2^ = 0.086) and 2-back condition (*F*(3,192) = 11.18, *p* < .001, *η*_*p*_^2^ = 0.149) but not in the control condition. Post-hoc tests revealed differences between pre- and mid-training (*Z* =  − 2.70, *p* = .007, *r* = 0.330 for 1-back; *Z* =  − 3.43, *p* = .001, *r* = 0.419 for 2-back), pre- and post-training (*Z* =  − 3.17, *p* = .002, *r* = 0.387 for 2-back) pre-training and FU (*Z* =  − 3.31, *p* = .001, *r* = 0.404 for 1-back; *Z* =  − 5.00, *p* < .001, *r* = 0.611 for 2-back) as well as between post-training and FU (*t*(66) = 2.76, *p* = .008, *d* = 0.337 for 1-back; *t*(66) = 2.81, *p* = .007, *d*_Cohen_ = 0.343 for 2-back).

As no false alarms occurred in the n-back task, only statistics of hits were calculated as analyses of both hits and misses would be redundant. No significant effects were found for the numbers of hits in 1-back and control conditions. However, mixed ANOVA revealed a significant main effect of time for the number of hits in the 2-back condition (*Greenhouse–Geisser* *F*(2.23,142.48) = 7.43, *p* = .001, *η*_*p*_^2^ = 0.104). Post-hoc tests revealed differences between pre- and post-training (*Z* = -2.95, *p* = .003, *r* = 0.360) as well as between pre-training and FU (*Z* = -3.94, *p* < .001, *r* = 0.481).

fNIRS data from one individual in the fNIRS group had to be excluded over the left dlPFC at intermediate assessment due to bad data quality. Mixed ANOVA revealed a main effect of group in the 1-back condition over the right dlPFC (*F*(2,64) = 4.16, *p* = .020, *η*_*p*_^2^ = 0.115. According to the post-hoc Tukey analysis, the fNIRS group was characterized by significantly lower dlPFC activation compared to the SCP group (0.45, *p* = .020). Moreover, there was a significant treatment-by-time interaction in the 1-back condition over left dlPFC (*F*(6,192) = 3.87, *p* = .001, *η*_*p*_^2^ = 0.108). Post-hoc Tukey HSD revealed a significantly lower left dlPFC activation in 1-back in the NIRS- compared to EMG group at FU (0.64, *p* = .049, g* = 0.783). There was a statistically significant increase of dlPFC activation in the EMG- (*F*(3,57) = 3.23, *p* = .029, *η*_*p*_^2^ = 0.145) and a statistically significant decline in the SCP group (*F*(3,75) = 5.21, *p* = .003, *η*_*p*_^2^ = 0.173). We found no significant effects for the 2-back condition on the neurophysiological level.

#### Go/NoGo task

Information on mean, standard deviation and range of behavioral data are available in the Supplements (Table S7). We found a significant main effect for group in correct trials’ reaction times (*F*(2,64) = 3.74, *p* = .029, *η*_*p*_^2^ = 0.105). Post-hoc Tukey HSD revealed significantly faster reaction times in the EMG- compared to the SCP group (32.47, p = .023). ANOVA revealed a significant main effect for time (*F*(3,192) = 5.50, *p* = .001, *η*_*p*_^2^ = 0.079) and group (*F*(2,64) = 3.17, *p* = .048; *η*_*p*_^2^ = 0.090) for the number of false alarms (and thereby for correct rejection in reverse direction). Results of paired samples post-hoc tests (Bonferroni-adjusted significance level α = 0.008) indicate that false alarms significantly decreased between pre- and post-training (*t*(66) = 3.18, *p* = .002, *d*_Cohen_ = 0.388) as well as pre-training and FU (*t*(66) = 2.65, *p* = .010, *d*_Cohen_ = 0.324). Post-hoc Tukey HSD revealed a higher false alarm rate (and significantly lower rate of correct rejections) in the EMG group when compared with the SCP group (1.48, *p* = .037). Post-hoc paired tests did not reveal any significant effects for time that survived Bonferroni correction.

Data from one individual (same as for n-back) in the fNIRS group had to be excluded at intermediate assessment due to bad data quality. Mixed ANOVA revealed no significant effect of dlPFC activation in the Go/NoGo task.

## Discussion

We investigated short and longer-term effects of SCP- and frontal fNIRS-NF compared to a semi-active EMG control training in an adult ADHD sample. Results indicate statistically significant improvements in primary and secondary clinical and neurocognitive measures over the training course in both active groups as well as in the semi-active control group. Improvements remained stable 6 months after training, suggesting long-lasting effects. Hence, we observed no superior effects for SCP- or fNIRS-NF in the overall sample. Only when separating learners from non-learners, a superior effect on self-rated global symptoms as well as hyperactivity and impulsivity emerges for learners when compared to non-learners or the semi-active EMG training with small to large effect sizes. The percentage of improved cases in terms of impulsivity in the fNIRS-NF group was higher compared to the other groups which shows that the counterintuitive fNIRS protocol (with increases in the feedback signal corresponding to cortical deactivation and decreases in the feedback signal reflecting cortical activation) did not adversely affect patients’ well-being compared to the other conditions. Our findings add to the findings of the earlier analyses of a sub-group of SCP participants^45,48^, particularly in terms of non-specific effects of NF that could not yet be investigated at that stage of data collection.

Regarding clinical effects induced by NF, our results are somehow in line with recent reports of sustained effects after NF in comparison with active and non-active control groups in children (e.g.,^49^) and adolescents with ADHD (e.g.,^26^). Our findings extend those from the only previously reported sham-controlled study investigating NF in adult patients with ADHD^50^. Although considering many of the limitations of earlier studies, this previous study could not show that adult patients with ADHD were able to learn self-regulation by means of the implemented neurofeedback protocol. Reward thresholds were automatically adjusted to provide positive feedback about 80% of the time. In the light of operant learning this could be problematic as at every reset of the reward threshold, patients were either rewarded for not learning to self-modulate the targeted parameter or were punished for successful learning. Furthermore, it has been shown that specificity is more important than sensitivity when learning brain self-regulation^51^. This might explain the lack of self-regulation and in consequence the lack of differences between NF and sham feedback on any outcome measure. In our study, we provide an indication that a proportion of adults with ADHD was able to progressively self-regulate the allocated target parameters (i.e., SCP, frontal hemodynamic response) across 30 training sessions although changes in amplitudes over the training were not statistically significant when looking at the whole sample. As can be seen from the plots depicting learning curves (Figs. 3, 4), there is huge variation in the data, indicating noticeable interindividual differences in performance. It has been argued in the literature that the benefits of NF are not exclusively caused by the neuromodulation that is in turn associated with specific changes in behavior, but may rather be mediated or moderated by non-specific effects such as feelings of self-efficacy, motivation or social interacting mechanisms^52^. We did not observe group differences in expectation, fit between therapist and patient, therapeutic relationship, persuasiveness of the therapist or willingness of the patient to engage over the training course. However, we found a significant increase regarding perceived therapist expertise over the training course in all groups, and fNIRS non-learners perceived the therapist as less competent as compared to fNIRS learners. Thus, the beneficial effects we found here in both active NF groups – but also in the semi-active control group – could, to a high amount, be due to other non-specific variables such as learning to focus on the task for a long period of time in a relatively monotone setting. Furthermore, the EMG-BF was relatively challenging and self-regulation mechanisms might have played a key-role in this group as well. There are older studies showing that EMG-BF leads to improvements in hyperkinetic symptoms^53^. An earlier publication analyzing EEG frequency data of individuals in the EMG group, however, did not reveal systematic effects induced by EMG-BF on brain activity^54^. Likewise, symptom improvements and cognitive improvements at follow-up may reflect regression to the mean. A meta-analysis by Emmert and colleagues^55^ showed that independent of the target ROI, individuals co-activate a cognitive control network presumably associated with self-regulation per se, independently of the self-regulated region. These regions in turn have been repeatedly reported to be underactivated in ADHD (cf., e.g.,^10^). This might lead to the conclusion that self-regulation itself leads to benefits in adult ADHD independent of the ROI. Yet, as we could demonstrate that learning and non-learning leads to differential outcome on the symptom level, it is quite likely that specific mechanisms contribute to the symptom improvements additionally to non-specific mechanisms but only for those who learn to regulate the target parameter. We observed no specific changes in the coupled behavior without learning neuroregulation.

During the first training phase in the fNIRS group – which unknowingly was confronted with a counterintuitive visual feedback – participants used strategies that led to a feedback curve on the screen that was not corresponding to the prompt (e.g., arrow pointing down but the feedback curve moved upwards, i.e., participants induced deactivation). After the break, this pattern was reversed. Now, participants regulated according to the visual feedback given on the screen. This underlines the central role of the feedback in NF (even when the instruction is counterintuitive and does not support “spontaneous” regulation efforts). In this unintended switch of polarization, participants might have started to rely on the feedback rather than the task to, e.g., “activate” when the arrow was pointing upwards. A similar but intended result was reported by Siniatchkin and colleagues^56^. After two sessions with successful regulation, healthy children received inverted feedback, i.e., they had to regulate in the opposite direction. Although children did not change their strategies, they again were successful after a short period of deterioration. The authors conclude that participants rely more on feedback and reinforcement than on (instructed) strategies. We cannot corroborate these assumptions with data though, as we did not systematically assess strategies. Thus, we can only make presumptions based on brain data assessed during the training.

We could not statistically analyze the impact of medication on learning or outcome due to respectively small sub-sample sizes. On a descriptive level, however, the relative amount of participants on medication in the SCP group was higher in those classified as learners as compared to those classified as non-learners. This was not the case in the fNIRS group. These results might suggest that the effects of fNIRS-NF are similar for medicated and unmedicated individuals but might not be similar in SCP-NF, but further studies in larger samples are necessary to corroborate this idea.

On an electrophysiological level, we found CNV amplitudes to differ at baseline with the EMG group providing the most negative CNV amplitudes (small effect size for fNIRS and large effect size for SCP) which later converged over the training course. As we did not find any other baseline differences concerning current symptom ratings, this baseline difference is hard to interpret. We found P300 amplitudes to decrease over the training course in all groups (medium effect size). This might suggest that each of the trainings modulate top-down processes of attention control and participants habituate processes of attention orienting requiring less attentional resources^57,58^. Our findings are concordant with those of Studer et al.^59^ who, in healthy adults, have found a pre-post decrease in P300 amplitudes after theta/beta NF.

With respect to hemodynamic responses in a WM task, we found right dlPFC activity to differ between groups in the low WM load condition. Moreover, we observed a differential development of amplitudes in the left dlPFC in the 1-back condition over time, with an increase of dlPFC activity in the EMG group and a decrease in the SCP group. Because of this differential effect, a mere habituation process seems improbable. Moreover, as we did not see such a differential effect in the high WM load condition or the behavioral data (reaction times decreased significantly over time independent of group allocation), we also do not interpret these findings as an actual improvement or decline in WM function; instead, we assume that different strategies were applied in the two groups, with a focus on effortful, executive control in the EMG group and possibly a progressively language-based strategy in the SCP group (which would not have involved the dlPFC as the main task-related area but instead the inferior frontal cortex; cf.^,46^), at least in the relatively easy 1-back condition. How these different strategies were directly or indirectly related to the respective training parameter remains, however, speculative.

On the behavioral level, reaction time decreased over the training course in the high- and low WM condition in all training groups and the number of hits in the high WM load condition equally increased in all groups. Both effects could be explainable by either cognitive improvements due to the training or – what is more probable as the improvement also occurs in the control group – by simple practice effects.

In the Go/NoGo task, on a behavioral level, reaction times decreased in all groups and the number of false alarms decreased whereas no changes were observed on the electrophysiological level. We did not investigate neurophysiological data separately for learners and non-learners due to small sample sizes in each of these subgroups and thereby statistical power problems in reliably detecting neurophysiological changes.

### Limitations

We applied an ANOVA to analyze learning because this was the intended analysis strategy published in the registration of the trial^32^. Even though, in retrospect, it is not the optimal method, we decided to adhere to the published approach. This analysis strategy is still helpful to gain an overview on how learning developed over time and to put the reported results into context (cf. CRED-nf checklist^60^), though no detailed insights can be obtained. Therefore, we aim to publish a follow-up paper with a detailed analysis of learning within and between sessions in the different groups using linear mixed models. Thus, having a closer look on how learning interacts with clinical outcome and how learning develops within and between sessions will give important insights into the mechanisms of NF in adults with ADHD.

One possible limitation of our fNIRS-NF implementation is that we used a common average reference (CAR). Previous fNIRS studies also have used a CAR to reduce global artifacts such as respiration, heartbeat or motion artifacts in the hemodynamic response (e.g.,^29^). However, the CAR bears the risk to punish actually beneficial network activity (see^61^). Yet, as Marx et al. could still demonstrate promising results after NF in children with ADHD, it is unlikely that the CAR eliminates all network activity. However, using a different algorithm might better support the learning process and result in better outcomes.

Another apparent limitation of the study is the lack of blinding, probably leading to expectancy effects on the part of participants as well as on the part of the investigators. Moreover, investigators might have had difficulties in maintaining a neutral position (over- or undercompensation when training individuals in the control condition). As the same investigators conducted the training sessions as well as the diagnostic interviews, the positive results obtained with the WRI have to be interpreted with caution.

## Conclusion

In conclusion, this study in adult patients with ADHD indicates that SCP- and frontal fNIRS NF are feasible but don’t lead to superior short- and longer-term effects compared to a semi-active control condition. Only when differentiating learners from non-learners, additional beneficial effects on symptom ratings became visible in the subgroup of learners. These findings support the assumption of NF as a neurobiological treatment approach with non-specific as well as specific modes of action associated with regulation abilities.

## Supplementary Information

Supplementary Information.
