# Author Correction: A randomized-controlled neurofeedback trial in adult attention-deficit/hyperactivity disorder

**DOI:** 10.1038/s41598-022-10840-6

**Published:** 2022-04-19

**Authors:** Beatrix Barth, Kerstin Mayer-Carius, Ute Strehl, Sarah N. Wyckoff, Florian B. Haeussinger, Andreas J. Fallgatter, Ann-Christine Ehlis

**Affiliations:** 1 Psychophysiology and Optical Imaging, Department of Psychiatry and Psychotherapy, University of Tübingen, Calwerstr. 14, 72076 Tübingen, Germany; 2 LEAD Graduate School and Research Network, University of Tübingen, Gartenstr. 29, 72074 Tübingen, Germany; 3 Department of Psychiatry and Psychotherapy, Tübingen Center for Mental Health (TüCMH), University of Tübingen, Calwerstr. 14, 72076 Tübingen, Germany; 4 Institute for Medical Psychology and Behavioural Neurobiology, University of Tübingen, Silcherstr. 5, 72076 Tübingen, Germany; 5 Werner Reichardt Centre for Integrative Neuroscience (CIN), University of Tübingen, Otfried-Müller-Str. 25, 72076 Tübingen, Germany; 6 Present Address: Faculty of Sport Science, Institute of Sport Psychology and Physical Education, Leipzig University, Jahnallee 59, 04109 Leipzig, Germany; 7 Present Address: Division of Pediatric Neurology, Barrow Neurological Institute at Phoenix Children’s Hospital, Phoenix, AZ USA

Correction to: *Scientific Reports* https://doi.org/10.1038/s41598-021-95928-1, published online 19 August 2021

The original version of this Article contained an error in Table 1 where row 2 was incorrect for “Characteristic”,

“Sex, female/male, no”

now reads:

“Sex, male/female, no”

The original Article has been corrected.

