# Supplementary material for: A randomized-controlled neurofeedback trial in adult attention-deficit/hyperactivity disorder
Source: Sci Rep. 2021 Aug 19;11:16873. doi: 10.1038/s41598-021-95928-1 (PMC8376871; doi:10.1038/s41598-021-95928-1)
Supplement: Supplementary file 1 — Supplementary Information. [file 41598_2021_95928_MOESM1_ESM.pdf]

A randomized-controlled neurofeedback trial in adult attention-deficit/hyperactivity disorder

Beatrix Barth [1, 2,3], Kerstin Mayer-Carius [4,5], Ute Strehl [4], Sarah N. Wyckoff [4, 6], Florian B. Haeussinger[1], Andreas J Fallgatter [1, 3, 7], Ann-Christine Ehlis [1, 2, 3]

Contents

1. Statistical analysis and preprocessing ……………………………..………………………………... 2

1.1. Slow cortical potential neurofeedback ……………………………..…………………………….. 2

1.2. Functional near-infrared spectroscopy neurofeedback ……………………………...…...….... 2

1.3. ERP outcome measures ……………………………..…………………………………......…..... 3

- *Go/NoGo task (CNV) ……………………………..…………………………….….... 3*
- *P300 acoustic counting task ……………………………..……………………………..... 3*

1.4. fNIRS outcome measures ……………………………..……………………..……………..…... 4

- *n-back task ……………………………..……………………………………........….…... 4*
- *Go/NoGo task ……………………………..…………………………………….…….... 4*

Table S1. Ratings of non-specific factors during over the training course ……………….……….......... 5

Figure S1. Changes in perceived expertise of the therapist over the training course…………….…...... 6

Table S2. Behavior ratings after half of the sessions, after feedback training and at 6-month follow-up for each group …………...…………………..……………………………………………….………….……….. 7

Figure S2. Clinical trajectories for BDI …………...…………………..…………………………….……….... 8

Table S3. Cognitive measures before feedback training, after feedback training and at 6-month follow-up for each group …………...…………………..…………………………………………….……………….. 8

Table S4. Reaction Time and errors in the Go/NoGo (CNV) task ………….…………………….……..…... 9

Table S5. Counting errors in the P300 task …………...…………………..…………………………….….. 9

Table S6. Reaction Time and errors in the n-back task …………...……………………….……….…….…... 10

Table S7. Reaction Time and errors in the Go/NoGo task …………...……………………………….….... 11

1. Statistical analysis and preprocessing

1.1. Slow cortical potential neurofeedback

The analyzed SCP data of the total sample were already analyzed and published in a sub-group of the total sample [1, 2]. We preprocessed electrophysiological data with the BrainVision Analyzer software Version 2.0 (Brain Products, Munich, Germany). We applied a low cutoff filter of 0.01 Hz and a 50 Hz notch filter. We segmented data for up- and down-regulation trials from 2000 ms pre-trial to 8000 ms after the start of each trial and baseline correction was performed 2000 ms pre-trial. We applied automatic artifact rejection (maximum allowed voltage step: 50 µV, maximum allowed absolute difference of two values: 150 µV, maximum/minimum allowed amplitude:150 µV/-150 µV; interval length 200 ms). We calculated the mean amplitude for the time interval 4–8 s after the start of each trial. We then calculated mean differences in amplitudes between SCP negativity minus positivity separately for the feedback and transfer condition. Thus, we report six measures for all sessions: negativity, positivity, and differentiation for the feedback as well as the transfer condition [1]. To estimate SCP self-regulation, the mean SCP amplitudes were averaged for sessions 2 + 3, 13 + 14, 17 + 18, 28 + 29 and 32 + 33 (aiming to deal with effects due to habituation or facing completion [1]). This analysis provides estimates of regulation performance and learning. A detailed analysis of self-regulation ability will be reported elsewhere (within- and across- session learning using linear mixed models including expectation and motivation factors). We analyzed SCP amplitude (µV) using task by session (five session averages) repeated-measures ANOVAs.

1.2. Functional near-infrared spectroscopy neurofeedback

We used MATLAB R2017a (MathWorks Inc, Natick, USA) for preprocessing of fNIRS data and Microsoft Excel 2014 and IBM SPSS Statistics version 26.0 for further statistical analyses. As part of offline data analysis and preprocessing, the fNIRS raw signal was processed using the temporal derivative distribution repair algorithm [TDDR; 3] to eliminate spike artifacts, and further processed with the correlation based signal improvement algorithm [4] to reduce movement artifacts. Subsequently, data were bandpass filtered (0.01–0.1 Hz) and channels that were strongly contaminated by different types of artifacts were interpolated (visual inspection). For further analysis

we focused only on corrected O_2Hb changes over left and right region of interest (ROI) channels. The task-related O_2Hb concentration changes were referred to a 5-s baseline interval prior to the regulation phase. Finally, we averaged O_2Hb amplitudes during the last 15 s of the regulation phase for the five session averages in activation and deactivation, respectively, for transfer and feedback. All further analyses of the O_2Hb amplitudes were identical to analyses for the SCP amplitudes.

1.3. ERP outcome measures

We preprocessed electrophysiological data with the BrainVision Analyzer software Version 2.0 (Brain Products, Munich, Germany).

Go/NoGo task (CNV)

We performed baseline correction on each segment (-700 ms relative to S1 until 3000 ms after S1). Segment-wise artifact rejection procedure semi-automatically removed segments with voltage steps exceeding 50 µV or exceeding absolute difference of two values of 200 µV, as well as amplitudes lower than −100 or higher than 100 µV, or segments containing lower activity than 0.5 µV. We calculated the mean CNV amplitude between 1000 and 1800 ms after S1. We analyzed the CNV amplitude for electrode site Fz exhibiting the most pronounced amplitude as calculated by a rank variance analysis. We calculated reaction time (RT) and response errors to the Go stimulus as measures of task performance [1, 5].

P300 acoustic counting task

We re-referenced EEG data to linked mastoids, and applied a 12-dB/octave Butterworth filter from 0.01 to 30 Hz as well as a 50-Hz notch filter. We segmented and baseline-corrected (200 ms pre-target) data for the Go trials 200 ms prior to the target tone until 700 ms after the target tone. Segment-wise artifact correction criteria were identical to CNV criteria. The P300 was identified by a semi-automatic peak detection procedure as the most positive peak in a time window ranging from 250 ms to 450 ms after the target tone. We analyzed the P300 amplitude for the electrode position Pz exhibiting the most pronounced amplitude as calculated by a rank variance analysis. Counting error as

measure of task performance was the absolute difference between the number of targets presented and the number as reported by the participants [1, 5].

1.4. fNIRS outcome measures

Preprocessing of the fNIRS raw signal was identical to the preprocessing steps for fNIRS-NF data. Behavioral data are reported in the Supplements.

n-back task

The difference in prefrontal activation between the high working memory (WM) load (2-back) and the control condition (0-back) as well as low WM minus 0-back served as neurophysiological measure of WM function. Data was averaged in left and right dlPFC (BA9 and BA46). ROI channels were selected according to previous studies [e.g., 6, 7]. Reaction times (RT), correct reactions (hits), omission errors, and false alarms were assessed.

Go/NoGo task

We calculated the activation difference between the NoGo minus Go conditions for frontal channels covering BA9, BA44, BA45 and BA46 [8, 9] as well as RT, correct reactions (hits), omission errors, correct rejections and false alarms during NoGo.

Table S1. Ratings of non-specific factors during over the training course

FERT subscale	Session	SCP	SCP learners	SCP non-learners	fNIRS	fNIRS learners	fNIRS non-learners	EMG
Expectation mean ± SD	5	39.22 ± 8.75	39.13 ± 8.05	39.26 ± 9.27	40.76 ± 8.53	42.77 ± 9.61	37.50 ± 5.45	40.97± 6.62
	10	38.38 ± 10.44	40.13 ± 8.44	37.61 ± 11.35	42.19 ± 8.79	46.00 ± 7.62	36.00 ± 7.11	42.70 ± 5.71
	15	38.69 ± 10.39	39.75 ± 11.63	38.22 ± 10.11	41.10 ± 8.19	44.46 ± 8.48	35.63 ± 3.58	43.66 ± 6.53
	20	39.35 ± 10.93	40.00 ± 12.82	39.06 ± 10.37	41.29 ± 5.78	42.54 ± 6.46	39.25 ± 4.03	39.80 ± 9.31
	25	39.76 ± 10.90	41.96 ± 12.33	38.78 ± 10.44	41.48 ± 7.89	44.85 ± 7.97	36.00 ± 3.70	41.30 ± 9.47
	30	39.96 ± 11.70	43.51 ± 13.58	38.39 ± 10.81	42.38 ± 9.61	44.62 ± 9.90	38.75 ± 8.43	41.56 ± 9.01
Fit mean ± SD	5	25.54 ± 2.66	24.75 ± 3.20	25.89 ± 2.40	25.19 ± 3.48	25.31 ± 2.83	25.00 ± 4.57	25.02± 3.39
	10	26.42 ± 1.88	25.63 ± 2.39	26.78 ± 1.56	25.81 ± 3.12	26.62 ± 2.76	24.50 ± 3.42	26.00 ± 3.15
	15	26.23 ± 2.01	26.63 ± 2.33	26.06 ± 1.89	26.62 ± 1.88	26.85 ± 1.86	26.25 ± 1.98	25.21 ± 4.26
	20	25.92 ± 2.97	25.25 ± 3.06	26.22 ± 2.96	25.93 ± 2.64	26.80 ± 1.71	24.50 ± 3.34	25.20 ± 3.40
	25	26.57 ± 2.19	26.62 ± 1.99	26.56 ± 2.33	26.24 ± 2.45	27.15 ± 1.14	24.75 ± 3.28	25.17 ± 4.11
	30	26.27 ± 2.27	26.37 ± 2.45	26.22 ± 2.26	26.43 ± 2.54	27.38 ± 0.87	24.88 ± 3.56	25.00 ± 3.43
Relation mean ± SD	5	50.42 ± 6.45	49.25 ± 9.91	50.94 ± 4.44	50.33 ± 6.43	50.54 ± 7.38	50.00 ± 4.96	51.21 ± 4.75
	10	50.62 ± 5.38	50.75 ± 4.92	50.56 ± 5.70	50.33 ± 6.41	51.31 ± 6.14	48.75 ± 6.92	51.90 ± 4.61
	15	51.15 ± 4.38	52.25 ± 4.86	50.67 ± 4.20	49.71 ± 5.69	50.54 ± 5.78	48.38 ± 5.63	51.23 ± 6.55
	20	51.23 ± 5.55	49.13 ± 8.51	52.17 ± 3.54	50.10 ± 5.91	51.62 ± 5.04	47.63 ± 6.70	51.45 ± 5.00
	25	52.18 ± 3.99	51.97 ± 5.06	52.28 ± 3.58	49.81 ± 6.71	50.85 ± 6.20	48.13 ± 7.57	52.33 ± 3.80
	30	51.76 ± 4.67	51.47 ± 4.16	51.89 ± 4.99	52.14 ± 4.03	52.85 ± 3.53	51.00 ± 4.75	52.10 ± 3.97
Expertise mean ± SD	5	22.85 ± 4.23	23.63 ± 4.63	22.50 ± 4.13	24.22 ± 2.63	25.13 ± 2.71	22.75 ± 1.75	22.89 ± 2.54
	10	23.42 ± 3.46	24.75 ± 2.61	22.83 ± 3.68	23.29 ± 4.40	25.08 ± 3.90	20.38 ± 3.66	23.35± 3.15
	15	24.38 ± 3.14	24.88 ± 3.09	24.17 ± 3.22	24.67 ± 3.28	26.23 ± 2.24	22.13 ± 3.18	24.63 ± 2.75
	20	23.88 ± 4.63	23.00 ± 6.78	24.28 ± 3.48	24.71 ± 3.38	26.38 ± 1.85	22.00 ± 3.63	25.05 ±2.59
	25	24.41 ± 3.94	23.58 ± 4.58	24.78 ± 3.70	24.12 ± 4.11	24.85 ± 4.36	23.00 ± 3.67	25.48 ± 2.19
	30	24.50 ± 4.12	24.39 ± 4.86	24.56 ± 3.90	24.90 ± 3.49	26.23 ± 1.64	22.75 ± 4.65	24.95 ± 2.63
Persuasiveness mean ± SD	5	25.42 ± 2.69	25.13 ± 2.95	25.56 ± 2.64	25.91 ± 2.58	26.78 ± 1.67	24.50 ± 3.25	26.12 ± 2.09
	10	25.27 ± 2.59	25.13 ± 2.70	25.33 ± 2.61	24.48 ± 3.54	25.08 ± 3.69	23.50 ± 3.30	26.05 ± 2.69
	15	25.88 ± 2.07	25.75 ± 2.55	25.94 ± 1.89	25.71 ± 3.68	26.92 ± 1.89	23.75 ± 5.04	26.15 ± 1.91
	20	25.77 ± 3.31	24.50 ± 5.13	26.33 ± 2.06	24.86 ± 4.50	25.92 ± 3.57	23.13 ± 5.52	26.40± 2.16
	25	25.78 ± 2.98	24.42 ± 4.20	26.39 ± 2.12	24.76 ± 3.70	25.00 ± 4.10	24.38 ± 3.16	26.41 ± 2.62
	30	26.06 ± 2.52	25.44 ± 3.50	26.33 ± 2.00	26.14 ± 3.02	26.38 ± 2.90	25.75 ± 3.37	28.90 ± 13.20
Willingness mean ± SD	5	13.77 ± 4.40	14.25 ± 3.28	13.56 ± 4.89	14.79 ± 4.09	15.13 ± 4.52	14.25 ± 3.50	13.20 ± 4.13
	10	14.85 ± 4.50	13.50 ± 6.23	15.44 ± 3.54	14.69 ± 3.70	15.81 ± 3.86	12.88 ± 2.75	15.83 ± 3.60
	15	14.58 ± 4.80	13.75 ± 5.45	14.94 ± 4.61	15.21 ± 4.25	16.23 ± 3.63	13.54 ± 4.88	15.50 ± 4.71
	20	16.04 ± 4.45	16.13 ± 6.28	16.00 ± 3.58	14.98 ± 3.60	16.05 ± 3.47	13.25 ± 3.28	14.65± 4.67
	25	15.54 ± 5.13	14.65 ± 5.78	15.94 ± 4.94	14.02 ± 4.82	14.19 ± 5.43	13.74 ± 3.98	16.47 ± 3.75
	30	15.13 ± 4.68	14.67 ± 5.39	15.33 ± 4.49	14.00 ± 7.11	14.54 ± 6.13	13.12 ± 8.87	15.85 ± 3.95

Note: FERT = German questionnaire for measuring common factors in psychotherapy[10]

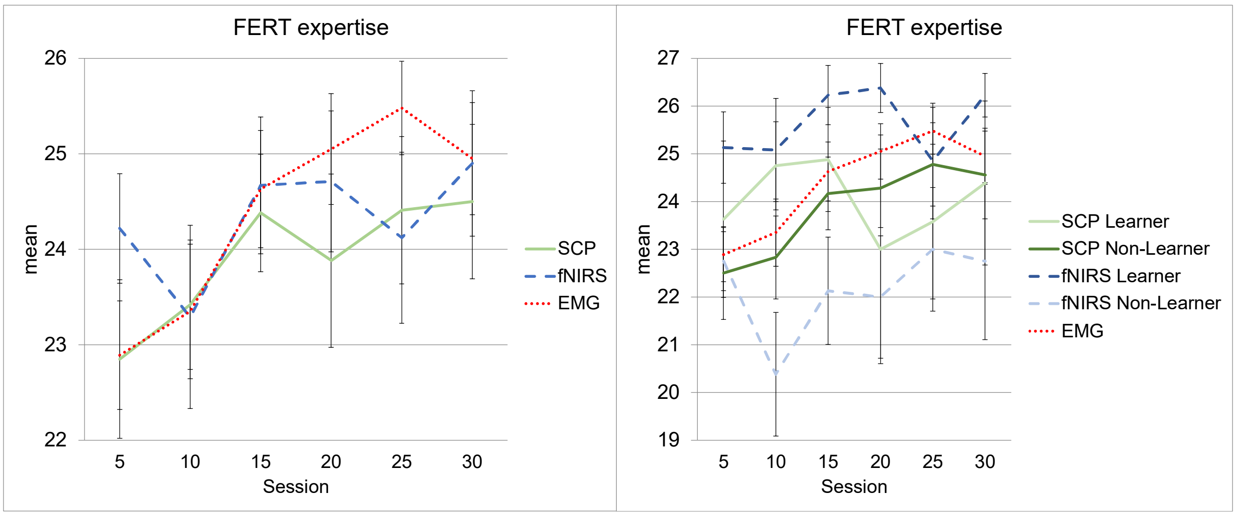

Figure S1. Changes in perceived expertise of the therapist over the training course

Table S2. Behavior ratings after half of the sessions, after feedback training and at 6-month follow-up for each group

	Mid<br>mean ± SD	Post<br>mean ± SD	FU<br>mean ± SD
**SCP**	**n = 26**	**n = 26**	**n =24**
**ADHS-SB Global**	24.88 ± 6.15	22.38 ± 6.42	22.49 ± 5.90
**ADHS-SB Inattention**	13.15 ± 4.58	11.73 ± 4.18	12.12 ± 3.67
**ADHS-SB Hyperactivity**	6.12 ± 1.758	5.46 ± 1.90	5.52 ± 2.21
**ADHS-SB Impulsivity**	5.62 ± 2.62	5.19 ± 2.51	4.85 ± 2.62
BDI	6.27 ± 6.44	6.00 ± 4.96	6.73 ± 5.57
**WRI Global**		31.96 ± 7.14	31.47 ± 6.72
**WRI Inattention**		7.12 ± 1.66	7.22 ± 1.77
**WRI Hyperactivity**		4.00 ± 1.17	4.26 ± 1.16
**WRI Impulsivity**		5.46 ± 2.16	5.42 ± 1.79
FEA current	21.02 ± 11.79	20.38 ± 9.15	16.08 ± 7.44
**NIRS**	**n = 21**	**n = 21**	**n = 21**
**ADHS-SB Global**	24.76 ± 8.54	21.52 ± 7.56	20.48 ± 7.94
**ADHS-SB Inattention**	13.52 ± 6.23	12.05 ± 5.52	11.14 ± 5.29
**ADHS-SB Hyperactivity**	6.19 ± 2.80	5.48 ± 2.27	5.14 ± 3.41
**ADHS-SB Impulsivity**	5.05 ± 2.78	4.00 ± 2.59	4.19 ± 2.40
BDI	6.38 ± 6.77	7.48 ± 7.92	5.67 ± 6.89
**WRI Global**		33.90 ± 8.32	33.97 ± 10.61
**WRI Inattention**		7.67 ± 2.31	6.84 ± 2.69
**WRI Hyperactivity**		4.24 ± 1.22	4.34 ± 1.31
**WRI Impulsivity**		5.48 ± 2.04	5.25 ± 2.71
FEA current	20.31 ± 9.64	22.06 ± 9.06	17.62 ± 4.21
**EMG**	**n = 20**	**n = 20**	**n = 20**
**ADHS-SB Global**	25.20 ± 7.34	23.60 ± 5.92	20.81 ± 9.03
**ADHS-SB Inattention**	13.20 ± 4.16	11.85 ± 3.53	10.45 ± 5.12
**ADHS-SB Hyperactivity**	6.55 ± 3.09	6.20 ± 3.09	5.59 ± 3.90
**ADHS-SB Impulsivity**	5.45 ± 2.40	5.55 ± 2.48	4.76 ± 2.99
BDI	7.45 ±7.59	6.05 ± 5.46	7.65 ± 7.98
**WRI Global**		33.20 ± 7.32	33.15 ± 9.99
**WRI Inattention**		7.20 ± 1.99	7.35 ± 1.69
**WRI Hyperactivity**		4.25 ± 1.16	4.60 ± 1.64
**WRI Impulsivity**		5.55 ± 2.46	6.40 ± 2.78
FEA current	*19.57 ± 8.59	+ 20.15 ± 8.15	§ 17.78 ± 5.93

Note: ADHS-SB = German ADHD self-rating scale for symptoms in adulthood [11, 12]; *BDI* = Beck-Depression Inventory [13]; *EMG* = Electromyography; *FU* = Follow-up; *SCP* = Slow Cortical Potential; *SD* = Standard Deviation; *WRI* = Wender-Reimherr-Interview [11, 12]: assessment at pre-, post and follow-up

* Information missing for 32 participants (46.3%)

+ Information missing for 37 participants (53.7%)

§ Information missing for 43 participants (62.7%)

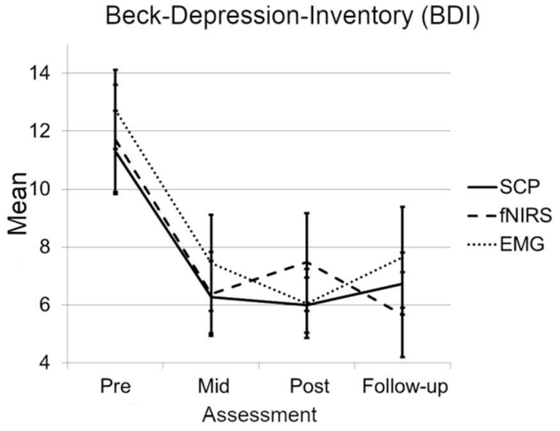

Figure S2. Clinical trajectories for BDI

Table S3. Cognitive measures before feedback training, after feedback training and at 6-month follow-up for each group

	Pre<br>mean ± SD	Post<br>mean ± SD	FU<br>mean ± SD
**SCP**	**n = 26**	**n = 26**	**n =24**
d2-R Concentration	104.19 ± 10.89	108.81 ± 11.48	109.75 ± 11.79
d2-R Operation Speed	103.62 ± 13.73	106.46 ± 12.60	108.63 ± 13.42
d2-R Accuracy	104.25 ± 10.70	107.15 ± 8.26	108.55 ±7.82
CFT-20-R	106.46 ± 19.82	118.13 ± 15.61	117.36 ± 15.39
**NIRS**	**n = 21**	**n = 21**	**n = 21**
d2-R Concentration	100.90 ± 12.02	105.67 ± 10.35	108.44 ±10.17
d2-R Operation Speed	102.10 ± 12.10	105.95 ± 11.88	108.58 ± 10.65
d2-R Accuracy	98.48 ± 10.36	105.76 ± 7.82	109.46 ± 11.20
CFT-20-R	112.62 ± 17.68	120.29 ± 18.03	121.55 ± 19.88
**EMG**	**n = 20**	**n = 20**	**n = 20**
d2-R Concentration	98.95 ± 11.14	103.70 ± 12.33	105.85 ± 12.12
d2-R Operation Speed	101.15 ± 14.63	105.60 ± 13.44	108.35 ±12.10
d2-R Accuracy	97.30 ± 13.23	103.00 ± 12.72	102.45 ± 15.19
CFT-20-R	110.50 ± 13.72	113.75 ± 13.28	117.50 ± 16.91

*Note: CFT-20-R = Culture Fair Test (*Weiss, 2006)*; d2-R =; EMG* = Electromyography; *FU* = Follow-up; *SCP* = Slow Cortical Potential; *SD* = Standard Deviation

* Information missing for 32 participants (46.3%)

$^+$ Information missing for 37 participants (53.7%)

§ Information missing for 43 participants (62.7%)

Table S4. Reaction Time and errors in the Go/NoGo (CNV) task

Assessment: **Group**	**RT Hit, ms, mean ± SD [range]**	**Hit, no, mean ± SD [range]**	**Miss, no, mean ± SD [range]**	**FA, no, mean ± SD [range]**	**Correct Rejection, no, mean ± SD [range]**
***Pre*:**					
**SCP**	665.08 ± 166.53 [474–1064]	47.73 ± 3.13 [34–49]	2.27 ± 3.13 [1–16]	2.15 ± 2.44 [1–13]	148.04 ± 1.61 [142–149]
**NIRS**	601.58 ± 94.59 [446–795]	48.81 ± 0.51 [47–49]	1.19 ± 0.51 [1–3]	2.19 ± 1.99 [1–9]	147.95 ± 1.53 [144–149]
**EMG**	575.14 ± 82.05 [435–728]	48.48 ± 1.37 [43–49]	1.52 ± 1.37 [1–7]	2.32 ± 2.65 [1–13]	148.00 ± 1.52 [143–149]
Mid:					
**SCP**	600.35 ± 134.36 [413–926]	48.65 ± 1.20 [43–49]	1.35 ± 1.20 [1–7]	2.50 ± 3.40 [1–18]	147.73 ± 2.78 [135–149]
**NIRS**	539.80 ± 79.27 [396–709]	48.85 ± 0.36 [48–49]	1.15 ± 0.36 [1–2]	2.05 ± 1.31 [1–6]	148.07 ± 1.24 [144–149]
**EMG**	546.27 ± 86.02 [413–771]	48.41 ± 1.96 [40–49]	1.59 ± 1.96 [1–10]	2.55 ± 2.39 [1–12]	147.59 ± 2.00 [140–149]
Post:					
**SCP**	591.50 ± 131.58 [415–922]	48.96 ± 0.20 [48–49]	1.04 ± 0.20 [1–2]	1.62 ± 0.80 [1–3]	148.38 ± 0.80 [147–149]
**NIRS**	546.12 ± 77.64 [433–702]	48.81 ± 0.51 [47–49]	1.19 ± 0.51 [1–3]	2.19 ± 2.11 [1–9]	148.00 ± 1.73 [144–150]
**EMG**	546.44 ± 76.07 [437–722]	47.85 ± 5.24 [25–49]	2.15 ± 5.24 [1–25]	1.88 ± 1.34 [1–6]	148.12 ± 1.34 [144–149]
FU:					
**SCP**	573.48 ± 128.97 [364–873]	47.73 ± 5.88 [19–49]	2.27 ± 5.88 [1–31]	1.56 ± 0.69 [1–3]	148.41± 0.69 [147–149]
**NIRS**	544.13 ± 78.42 [442–761]	48.86 ± 0.48 [47–49]	1.14 ± 0.48 [1–3]	2.00 ± 1.34 [1–6]	148.24 ± 1.00 [146–149]
**EMG**	546.04 ± 87.32 [428–801]	47.76 ± 3.60 [34–49]	2.24 ± 3.60 [1–16]	2.14 ± 1.49 [1–5]	147.87 ± 1.49 [145–149]

Note: EMG = Electromyography; *FA* = False Alarms; *FU* = Follow-up; *RT* = Reaction Time; *SCP* = Slow Cortical Potential; *SD* = Standard Deviation

Table S5. Counting errors in the P300 task

**Group**	**Pre mean, no, ± SD [range]**	**Mid mean, no, ± SD [range]**	**Post mean, no, ± SD [range]**	**FU mean, no, ± SD [range]**
SCP	3.99 ± 5.53 [0–17]	3.50 ± 5.32 [0–22]	1.87 ± 3.28 [0–13]	1.41 ± 1.82 [0–8]
NIRS	2.48 ± 4.59 [0–19]	1.35 ± 2.60 [0–11]	1.89 ± 3.02 [0–11]	2.28 ± 4.10 [0–15]
EMG	1.45 ± 2.39 [0–10]	1.32 ± 1.70 [0–6]	2.43 ± 4.41 [0–15]	2.10 ± 3.49 [0–14]

Note: *EMG* = Electromyography; *FA* = False Alarms; *FU* = Follow-up; *RT* = Reaction Time; *SCP* = Slow Cortical Potential; *SD* = Standard Deviation

Table S6. Reaction Time and errors in the n-back task

Assessment: Group	RT, ms, mean ± SD [range]	RT 1b, ms, mean ± SD [range]	RT 2b, ms, mean ± SD [range]	RT 0b, ms, mean ± SD [range]	Hit 1b, no, mean ± SD [range]	Hit 2b, no, mean ± SD [range]	Hit 0b, no, mean ± SD [range]	Miss 1b, no, mean ± SD [range]	Miss 2b, no, mean ± SD [range]	Miss 0b, no, mean ± SD [range]	FA 1b, no, mean ± SD [range]	FA 2b, no, mean ± SD [range]	FA 0b, no, mean ± SD [range]
Pre: **SCP**	534.77 ± 81.87 [389–663]	529.73 ± 100.42 [370–783]	596.85 ± 115.36 [421–853]	478.54 ± 80.51 [343–647]	11.92 ± 0.27 [11–12]	10.73 ± 1.71 [5–12]	11.88 ± 0.33 [11–12]	0.08 ± 0.27 [0–1]	1.27 ± 1.71 [0–7]	0.12 ± 0.33 [0–1]	0 ± 0 [0–0]	0 ± 0 [0–0]	0 ± 0 [0–0]
**NIRS**	540.19 ± 105.69 [410–775]	538.33 ± 126.89 [398–814]	597.52 ± 123.37 [472–902]	480.52 ± 89.65 [355–669]	11.52 ± 1.08 [8–12]	10.76± 2.10 [3–12]	11.71 ± 0.90 [8–12]	0.48 ± 1.08 [0–4]	1.24 ±2.10 [0–9]	0.29 ± 0.90 [0–4]	0 ± 0 [0–0]	0 ± 0 [0–0]	0 ± 0 [0–0]
**EMG**	487.85 ± 74.46 [369–676]	486.75± 84.10 [363–689]	556.45 ± 128.18 [358–779]	428.80 ± 60.80 [345–563]	11.80 ± 0.70 [9–12]	10.55 ± 2.86 [2–12]	11.85 ± 0.37 [11–12]	0.20 ± 0.70 [0–3]	1.45 ± 2.86 [0–10]	0.15 ± 0.37 [0–1]	0 ± 0 [0–0]	0 ± 0 [0–0]	0 ± 0 [0–0]
Mid: **SCP**	514.38 ± 101.41 [368–779]	506.57 ± 115.22 [365–796]	573.01 ± 137.78 [338–864]	468.84 ± 87.94 [350–716]	11.84 ± 0.47 [10–12]	11.05 ± 1.87 [3–12]	11.99 ± 0.05 [12–12]	0.16 ± 0.47 [0–2]	0.95 ± 1.87 [0–9]	0.01 ± 0.05 [0–0]	0 ± 0 [0–0]	0 ± 0 [0–0]	0 ± 0 [0–0]
**NIRS**	508.52 ± 80.92 [402–683]	496.24 ± 79.50 [362–592]	542.90 ± 105.52 [374–845]	475.71 ± 85.95 [362–712]	11.81 ± 0.68 [9–12]	11.29 ± 1.15 [8–12]	11.86 ± 0.36 [11–12]	0.19 ± 0.68 [0–3]	0.71 ± 1.15 [0–4]	0.14 ± 0.36 [0–1]	0 ± 0 [0–0]	0 ± 0 [0–0]	0 ± 0 [0–0]
**EMG**	458.75 ± 58.80 [366–575]	458.45 70.93 [362–592]	486.30 ± 89.48 [360–707]	430.05 ± 38.68 [352–490]	11.50 ± 0.69 [10–12]	11.10 ± 2.00 [5–12]	11.90 ± 0.31 [11–12]	0.50 ± 0.69 [0–2]	0.90 ± 2.00 [0–7]	0.10 ± 0.31 [0–1]	0 ± 0 [0–0]	0 ± 0 [0–0]	0 ± 0 [0–0]
Post: **SCP**	520.46 ± 110.70 [367–735]	509.50 ± 123.11 [357–749]	573.15 ± 143.77 [374–970]	469.65 ± 98.94 [334–738]	11.81 ± 0.49 [10–12]	11.46 ± 1.33 [6–12]	11.92 ± 0.39 [10–12]	0.19 ± 0.50 [0–2]	0.54 ± 1.33 [0–6]	0.08 ± 0.39 [0–2]	0 ± 0 [0–0]	0 ± 0 [0–0]	0 ± 0 [0–0]
**NIRS**	498.52 ± 92.18 [337–669]	492.38 ± 99.70 [321–662]	522.48 ±123.61 [348–833]	464.14 ± 77.61 [318–648]	11.48 ± 0.81 [9–12]	11.67 ± 0.58 [10–12]	11.67 ± 0.73 [9–12]	0.52 ± 0.81 [0–3]	0.33 ± 0.58 [0–2]	0.33 ± 0.73 [0–3]	0 ± 0 [0–0]	0 ± 0 [0–0]	0 ± 0 [0–0]
**EMG**	503.45 ± 110.77 [364–788]	516.05 ± 129.63 [342–794]	521.90 ± 130.08 [342–807]	467.40 ± 104.88 [373–865]	11.85 ± 0.37 [11–12]	11.00 ± 1.84 [5–12]	11.95 ± 0.22 [11–12]	0.15 ± 0.37 [0–1]	1.00 ± 1.84 [0–7]	0.05 ± 0.22 [0–1]	0 ± 0 [0–0]	0 ± 0 [0–0]	0 ± 0 [0–0]
FU: **SCP**	498.59 ± 96.08 [371–769]	490.74 ± 102.21 [352–710]	539.09 ± 145.94 [360–1031]	464.50 ± 87.04 [351–666]	11.86 ± 0.48 [10–12]	11.60 ± 1.38 [6–12]	11.95 ±0.20 [11–12]	0.14 ± 0.48 [0–2]	0.40 ± 1.38 [0–6]	0.05 ± 0.20 [0–1]	0 ± 0 [0–0]	0 ± 0 [0–0]	0 ± 0 [0–0]
**NIRS**	464.38 ± 63.83 [355–632]	466.71 ± 66.09 [366–660]	484.05 ± 89.38 [354–692]	440.00 ± 51.82 [344–544]	11.76 ± 0.54 [10–12]	11.67 ± 0.97 [8–12]	11.81 ± 0.51 [10–12]	0.24 ± 0.54 [0–2]	0.33 ± 0.97 [0–4]	0.19 ± 0.51 [0–2]	0 ± 0 [0–0]	0 ± 0 [0–0]	0 ± 0 [0–0]
**EMG**	470.40 ± 73.02 [336–595]	474.30 ± 79.79 [323–615]	494.30 ± 105.73 [324–705]	429.70 ± 51.97 [336–520]	11.60 ± 0.82 [9–12]	11.15 ± 1.87 [4–12]	11.75 ± 0.72 [9–12]	0.40 ± 0.82 [0–3]	0.85 ± 1.87 [0–8]	0.25 ± 0.72 [0–3]	0 ± 0 [0–0]	0 ± 0 [0–0]	0 ± 0 [0–0]

Note: *1b* = 1-back; *2b* = 2-back; *0b* = 0-back; *EMG* = Electromyography; *FA* = False Alarms; *FU* = Follow-up; *RT* = Reaction Time; *SCP* = Slow Cortical Potential; *SD* = Standard Deviation

Table S7. Reaction Time and errors in the Go/NoGo task

Assessment: Group	RT Hit, ms, mean ± SD [range]	RT Hit Control Condition, ms, mean ± SD [range]	Hit, no, mean ± SD [range]	Miss, no, mean ± SD [range]	FA, no, mean ± SD [range]	Correct Rejection, no, mean ± SD [range]	Hit Control Condition, no, mean ± SD [range]	Miss Control Condition, no, mean ± SD [range]
***Pre*:**								
**SCP**	465.25 ± 42.21 [386–574]	318.38 ± 41.50 [258–471]	31.52 ± 1.54 [30–32]	0.19 ± 0.49 [0–2]	3.33 ± 2.01 [0–8]	28.67 ± 2.01 [24–32]	63.12 ± 1.51 [57–64]	0.88 ± 1.51 [0–7]
**NIRS**	446.10 ± 46.93 [349–524]	296.29 ± 36.27 [245–375]	31.52 ± 1.54 [25–32]	0.48 ± 1.54 [0–7]	4.81 ± 3.12 [0–13]	27.19 ± 3.11 [19–32]	62.86 ± 1.77 [57–64]	1.14 ± 1.77 [0–7]
**EMG**	430.40 ± 40.80 [363–513]	295.55 ± 40.14 [221–353]	31.40 ± 1.50 [26–32]	0.60 ± 1.50 [0–6]	5.90 ± 3.14 [1–11]	26.10 ± 3.14 [21–31]	62.95 ± 1.91 [56–64]	1.05 ± 1.91 [0–8]
Mid:								
**SCP**	474.04 ± 60.97 [404–626]	314.62± 41.49 [257–442]	31.69 ± 0.84 [29–32]	0.31 ± 0.84 [0–3]	3.50 ± 1.84 [1–7]	27.42 ± 1.90 [25–31]	63.00 ± 1.96 [54–64]	1.00 ± 1.96 [0–10]
**NIRS**	456.52 ± 40.21 [380–515]	304.52± 55.64 [208–447]	31.71 ± 0.72 [29–32]	0.29 ± 0.72 [0–3]	3.71 ± 1.90 [1–7]	28.29 ± 1.90 [25–31]	62.71 ± 1.71 [58–64]	1.29 ± 1.71 [0–6]
**EMG**	433.65 ± 41.22 [367–534]	282.55 ± 31.85 [225–358]	31.90 ± 0.31 [31–32]	0.10 ± 0.31 [0–1]	3.95 ± 2.80 [0–11]	28.05 ± 2.80 [21–32]	62.85 ± 1.63 [57–64]	1.15 ± 1.63 [0–7]
Post:								
**SCP**	471.27 ± 58.14 [370–638]	310.12 ± 33.86 [255–394]	31.77 ± 0.82 [28–32]	0.23 ± 0.82 [0–4]	2.81 ± 1.98 [0–7]	29.19 ± 1.98 [25–32]	62.96 ± 1.18 [59–64]	1.04 ± 1.18 [0–5]
**NIRS**	463.33 ± 61.03 [359–584]	301.00 ± 50.36 [212–383]	31.33 ± 1.62 [25–32]	0.67 ± 1.62 [0–7]	3.14 ± 2.58 [0–8]	28.86 ± 2.58 [24–32]	62.90 ± 1.92 [56–64]	1.10 ± 1.92 [0–8]
**EMG**	447.70 ± 49.10 [355–572]	305.30 ± 43.75 [241–419]	31.40 ± 1.82 [24–32]	0.60 ± 1.82 [0–8]	4.10 ± 3.48 [0–15]	27.90 ± 3.48 [17–32]	62.95 ± 1.64 [58–64]	1.05 ± 1.64 [0–6]
FU:								
**SCP**	470.63 ± 54.04 [383–615]	314.40 ± 42.53 [253–444]	31.49 ± 1.03 [28–32]	0.51 ± 1.03 [0–4]	2.90 ± 2.50 [0–11]	29.10 ± 2.51 [21–32]	63.30 ± 1.10 [59–64]	0.70 ± 1.10 [0–5]
**NIRS**	446.43 ± 32.38 [395–513]	289.86 ± 35.23 [213–357]	31.90 ± 0.30 [31–32]	0.10 ± 0.30 [0–1]	3.43 ± 2.79 [0–10]	28.57 ± 2.79 [22–32]	62.90 ± 2.02 [55–64]	1.10 ± 2.02 [0–9]
**EMG**	439.55 ± 34.29 [392–520]	297.70 ± 45.15 [229–417]	31.50 ± 1.79 [24–32]	0.50 ± 1.79 [0–8]	4.50 ± 3.36 [0–12]	27.50 ± 3.36 [20–32]	62.70 ± 1.49 [59–64]	1.30 ± 1.49 [0–5]

Note: *EMG* = Electromyography; *FA* = False Alarms; *FU* = Follow-up; *RT* = Reaction Time; *SCP* = Slow Cortical Potential; *SD* = Standard Deviati

1. Mayer, K., Blume, F., Wyckoff, S.N., Brokmeier, L.L. & Strehl, U. Neurofeedback of slow cortical potentials as a treatment for adults with Attention Deficit-/Hyperactivity Disorder. *Clinical Neurophysiology*. **127**(2), 1374-1386 (2016).
2. Mayer, K., Wyckoff, S.N., Schulz, U. & Strehl, U. Neurofeedback for Adult Attention-Deficit/Hyperactivity Disorder: Investigation of Slow Cortical Potential Neurofeedback—Preliminary Results. *Journal of Neurotherapy*. **16**(1), 37-45 (2012).
3. Fishburn, F.A., Ludlum, R.S., Vaidya, C.J. & Medvedev, A.V. Temporal Derivative Distribution Repair (TDDR): A motion correction method for fNIRS. *NeuroImage*. **184**, 171-179 (2019).
4. Cui, X., Bray, S. & Reiss, A.L. Functional near infrared spectroscopy (NIRS) signal improvement based on negative correlation between oxygenated and deoxygenated hemoglobin dynamics. *NeuroImage*. **49**(4), 3039-3046 (2010).
5. Barth, B., et al. Identification of neurophysiological biotypes in attention deficit hyperactivity disorder. *Psychiatry and clinical neurosciences*. **72**(11), 836-848 (2018).
6. Ehlis, A.-C., Bähne, C.G., Jacob, C.P., Herrmann, M.J. & Fallgatter, A.J. Reduced lateral prefrontal activation in adult patients with attention-deficit/hyperactivity disorder (ADHD) during a working memory task: a functional near-infrared spectroscopy (fNIRS) study. *Journal of psychiatric research*. **42**(13), 1060-1067 (2008).
7. Fishburn, F., Norr, M., Medvedev, A. & Vaidya, C. Sensitivity of fNIRS to cognitive state and load. *Frontiers in Human Neuroscience*. **8**(76) (2014).
8. Monden, Y., et al. Individual classification of ADHD children by right prefrontal hemodynamic responses during a go/no-go task as assessed by fNIRS. *NeuroImage: Clinical*. **9**, 1-12 (2015).
9. Herrmann, M.J., Plichta, M.M., Ehlis, A.-C. & Fallgatter, A.J. Optical topography during a Go–NoGo task assessed with multi-channel near-infrared spectroscopy. *Behavioural Brain Research*. **160**(1), 135-140 (2005).
10. Vollmann, K., *Entwicklung und Überprüfung eines Fragebogens zur Erfassung relevanter Therapiebedingungen (FERT) [Dissertation].* , in *Mathematisch-Naturwissenschaftliche Fakultät* 2009, Eberhard-Karls-Universität Tübingen: Tübingen.
11. Rösler, M., Retz-Junginger, P., Retz, W. & Stieglitz, R. HASE–Homburger ADHS-Skalen für Erwachsene. *Göttingen: Hogrefe* (2008).
12. Rösler, M., et al. Tools for the diagnosis of attention-deficit/hyperactivity disorder in adults. Self-rating behaviour questionnaire and diagnostic checklist. *Der Nervenarzt*. **75**(9), 888-895 (2004).
13. Hautzinger, M., Keller, F. & Kühner, C., *Beck depressions-inventar (BDI-II)*. 2006: Harcourt Test Services Frankfurt.
